# Awakening Recovery: Enhancing Orexinergic Tone After Acute CNS Damage

**DOI:** 10.3390/ph18121879

**Published:** 2025-12-11

**Authors:** Paloma Otero-López, Xavier Madrid-González, Víctor Fernández-Dueñas, África Flores

**Affiliations:** 1 Pharmacology Unit, Department of Pathology and Experimental Therapeutics, School of Medicine and Health Sciences, Institute of Neurosciences, Universitat de Barcelona, 08907 Barcelona, Spain; palomaoterolopez@ub.edu (P.O.-L.); vfernandez@ub.edu (V.F.-D.); 2 Neuropharmacology and Pain Group, Neuroscience Program, IDIBELL-Bellvitge Institute for Biomedical Research, 08907 Barcelona, Spain

**Keywords:** orexin/hypocretin, acute CNS injury, neuroprotection, arousal, neurorehabilitation, spinal cord injury, stroke, traumatic brain injury, orexin receptor agonists

## Abstract

Acute injuries to the central nervous system (CNS) share a rapid disruption of arousal, autonomic stability, and neuroimmune balance. Among the neuromodulatory systems affected, the orexin (hypocretin) network is uniquely positioned at the intersection of wakefulness, autonomic control, and motivated behavior. Experimental evidence across ischemic, hemorrhagic, traumatic, and systemic models shows that orexin signaling is sharply suppressed during the early post-injury collapse and gradually recovers as arousal circuits and homeostatic functions stabilize. Controlled enhancement of orexinergic tone has been found to improve arousal state, modulate inflammatory responses, and support behavioral engagement, although these effects are highly dependent on timing, receptor subtype, and physiological context. This review synthesizes evidence from ischemia, hemorrhagic stroke, traumatic brain and spinal cord injury, and systemic inflammatory states, and examines the conceptual and translational rationale for targeting orexin pathways. We summarize available pharmacological, peptide-based, neuromodulatory, and physiological strategies to boost orexinergic tone, highlighting the growing development of selective OX_2_ agonists and experimental approaches to enhance endogenous orexin activity. By integrating findings across etiologies within a timing-aware framework, this review addresses a gap in the current literature, which has largely treated these injuries in isolation. While clinical testing in acute CNS injury has not yet been performed, the mechanistic convergence across etiologies suggests that orexinergic modulation may offer a phase-sensitive means to stabilize arousal and support recovery. Taken together, orexin emerges as a state-dependent integrator whose modulation could complement existing therapies by linking early arousal stabilization with longer-term motivational and functional recovery.

## 1. Introduction

Acute central nervous system (CNS) damage, whether caused by stroke, trauma, cardiac arrest, or systemic inflammation, remains among the most devastating medical emergencies, with few therapies capable of altering long-term outcomes. The initial mechanical or vascular insult is only the beginning. What follows is a cascade of neurovascular dysfunction, energy failure, autonomic instability, and maladaptive inflammation that often extends far beyond the primary lesion [1,2,3]. Despite advances in reperfusion, neurocritical care, and rehabilitation, survivors often face persistent cognitive, motor, and motivational deficits. These limitations reflect not only structural loss but a sustained failure of neuromodulatory systems that coordinate arousal, metabolism, and adaptive behavior [4].

Among these systems, the orexin (hypocretin) network of the lateral hypothalamus has emerged as a surprisingly powerful regulator of recovery potential. Originally characterized for its role in maintaining wakefulness, orexin signaling also links metabolic status to autonomic control, emotion, and goal-directed action [5,6,7]. Through widespread projections to monoaminergic, cholinergic, and motor circuits, orexin neurons sustain not just vigilance, but the capacity to mobilize effort and purposeful movement. These integrative properties place the orexin system at the intersection of nearly every process disrupted after acute CNS injury: disordered arousal, autonomic instability, impaired motivation, and compromised circuit engagement.

This perspective recasts orexin not as a sleep peptide, but as a homeostatic integrator of survival that synchronizes energy availability with behavioral demand. Its dysfunction may therefore explain a wide spectrum of post-injury states, from coma and hypoarousal to fatigue and apathy. Conversely, controlled reactivation of orexin pathways has been shown experimentally to stabilize arousal and dampen inflammatory responses, raising the possibility that orexinergic modulation could help the injured CNS transition from early physiological collapse to more coordinated recovery. Recent progress on both peptide-based and small-molecule orexin receptor agonists [8] has turned a once theoretical idea into a tangible pharmacological strategy, renewing interest in its translational potential across acute and chronic neurorepair.

Here, we review current evidence linking orexin signaling to recovery from acute CNS damage, from pathophysiological foundations to translational strategies. We outline the shared biological cascades that underlie these conditions, summarize how orexin integrates cellular, autonomic, and motivational responses to injury, and examine experimental and clinical interventions that enhance orexinergic tone. Although previous reviews have typically focused on single etiologies, such as traumatic brain injury, stroke, or narcolepsy, none have synthesized orexin biology within a unified, timing-aware framework that spans acute vascular, traumatic, spinal, and systemic insults. By embedding orexin mechanisms into the conserved temporal logic of acute CNS injury, this review aims to clarify when and how orexin modulation may be beneficial or potentially harmful, highlight conceptual blind spots in the current literature, and define the practical challenges that must be resolved for orexin-based therapies to progress from laboratory insight to clinical reality.

## 2. Methods

This work was designed as a narrative and mechanism-focused review. Literature was gathered through iterative searches conducted between August and November 2025 using PubMed, Google Scholar, and the AI-assisted tool Elicit. Elicit was used to broaden retrieval and reduce the chance of missing relevant studies, especially across different injury etiologies. Search terms combined “orexin” OR “hypocretin” with “stroke”, “ischemia”, “intracerebral hemorrhage”, “subarachnoid hemorrhage”, “traumatic brain injury”, “spinal cord injury”, “sepsis”, “cardiac arrest”, “encephalopathy”, “fatigue”, “arousal”, “neuromodulation”, “intranasal”, “OX1R”, “OX2R”, “danavorexton”, “TAK-925”, “TAK-861”, and “ALKS-2680”, among others. We included preclinical and clinical studies, reviews, and recent conference abstracts when they contained essential information not yet available in peer-reviewed publications (particularly for emerging OX_2_ agonists). Although English-language articles were prioritized, we included one study published in Chinese because it provided the only available preclinical data for spinal cord injury (SCI) within this context. Reference lists from key articles were also examined to identify additional studies not captured in keyword searches. Because this is a narrative rather than systematic review, no formal risk-of-bias tool or structured quality assessment was applied. Instead, the synthesis reflects an integrative and contextual appraisal of the literature, with attention to recurring methodological constraints highlighted in the field (e.g., small sample sizes, single-lab findings, limited follow-up, use of comorbidity-free rodent models, or lack of replication). This approach aimed to balance breadth and mechanistic depth while maintaining transparency about the heterogeneity and limitations of the available evidence.

## 3. Pathophysiology of Acute CNS Damage: Common Cascades and Points of Divergence

### 3.1. Common Temporal Sequence Across Etiologies

Acute insults to the CNS arise from distinct causes, including vascular occlusion or rupture in stroke, mechanical impact in traumatic brain or spinal injury, or global ischemia and systemic inflammatory crises. Despite etiological differences, they all converge on a remarkably similar biological logic. Each begins with a primary event that abruptly disrupts perfusion or tissue integrity, followed by secondary cascades that propagate damage and shape recovery potential. Immediately, loss of perfusion or mechanical disruption precipitates energy failure, ionic disequilibrium, and excitotoxic glutamatergic drive, triggering necrotic and apoptotic cell death [4,9]. Within hours to days, blood–brain and blood–spinal cord barrier dysfunction, microvascular failure, neuroinflammation, and evolving edema amplify the lesion. Over subsequent weeks, glial and stromal remodeling forms a compartmentalized injury border and fibrotic core [10,11]. Circuit-level adaptations and plasticity then determine the balance between persistent deficits and compensation. This shared backbone underlies heterogeneous clinical entities and sets the stage for targeted, timing-sensitive therapies.

### 3.2. Cardiovascular Etiologies: Focal Arterial Occlusion, Parenchymal Bleeding, and Whole-Brain Ischemia

#### 3.2.1. Ischemic Stroke

In ischemic stroke, abrupt arterial occlusion produces a core–penumbra architecture: an irreversibly injured core surrounded by hypoperfused, electrically silent but potentially salvageable tissue, whose outcome depends on reperfusion timing and collateral flow [9]. The biological substrate is rapid ATP depletion, membrane depolarization, Na^+^/Ca^2+^ influx, and excitotoxicity, followed by oxidative stress, mitochondrial injury, and microvascular failure. Reperfusion can rescue penumbra but it also introduces reperfusion injury and inflammatory amplification. Clinically, workflow aims to restore perfusion while limiting edema and hemorrhagic transformation [12]. Blood–brain barrier (BBB) dysfunction is an early and dynamic feature, driven by tight-junction alterations, increased transcytosis, pericyte changes, and endothelial inflammatory signaling. It not only contributes to edema formation but also critically influences drug delivery strategies [11].

#### 3.2.2. Intracerebral Hemorrhage (ICH)

In ICH, primary parenchymal bleeding abruptly elevates local pressure and shears tissue. Early outcomes are dominated by hematoma expansion, which is influenced by hypertension and anticoagulation status. Secondary injury reflects perihematomal edema, iron toxicity, and sterile inflammation around the clot [13]. Care bundles that couple rapid blood pressure reduction, urgent anticoagulant reversal when indicated, and specialist stroke-unit care improve survival. Minimally invasive evacuation strategies are being refined, though the impact on functional outcomes remains under active evaluation [14]. The underlying pathophysiology remains a blend of mass effect, BBB injury, and innate immune activation, with timing critical for each countermeasure.

#### 3.2.3. Subarachnoid Hemorrhage (SAH) and Global Ischemia After Cardiac Arrest

Although SAH originates from a focal vascular rupture, extravasated blood rapidly disseminates through the cerebrospinal fluid (CSF), triggering diffuse vasospasm, metabolic failure, and impaired arousal. Consequently, its secondary cascades resemble those of global hypoxic–ischemic injury rather than purely focal stroke [12]. This hybrid nature justifies grouping SAH alongside post-cardiac arrest brain injury. Both share a prominent microvascular and inflammatory signature superimposed on diffuse ischemic stress [15]. Following return of spontaneous circulation, the brain experiences a post-resuscitation syndrome marked by diffuse edema, excitotoxicity, and impaired autoregulation; targeted temperature and perfusion management are designed around these mechanisms [16]. In SAH, delayed cerebral ischemia from vasospasm and BBB failure drives secondary deterioration on top of the hemorrhagic insult. The unifying thread is global ischemic and inflammatory stress in a brain with fragile hemodynamics.

### 3.3. Traumatic CNS Injury: Mechanical Initiation, Biochemical Propagation

#### 3.3.1. Traumatic Brain Injury (TBI)

TBI begins with mechanical forces (linear/angular acceleration, impact, blast) producing focal contusions and diffuse axonal injury. The primary physical insult is rapidly followed by ionic flux, excitotoxic neurotransmission, mitochondrial dysfunction, and metabolic crisis, all of which broaden tissue loss [4]. Cerebrovascular dysfunction and BBB failure promote vasogenic edema, while sterile inflammation recruits microglia, infiltrating leukocytes, and cytokine networks that can be both reparative and damaging [17]. The field now recognizes TBI as a multisystem disorder of the neurovascular unit and immune network, not only of neurons and axons, which explains the variability in trajectories and the potential for targeted interventions beyond pure neuroprotection. During the subacute phase, astrocyte-rich borders and a fibrotic stromal core form to contain damage. These structures, once termed a “glial scar,” are now viewed as coordinated compartments that regulate axon guidance and immune traffic [10,11]. Understanding when to modulate versus harness these barriers is crucial for designing interventions.

#### 3.3.2. Spinal Cord Injury (SCI)

SCI mirrors TBI’s two-stage logic: a primary mechanical insult (contusion, compression, laceration) followed by secondary injury comprising ischemia, ionic dysregulation, excitotoxicity, free radical stress, edema, and robust inflammation [18]. Vascular compromise at and around the lesion and barrier breakdown extend tissue loss beyond the impact site. Over time, the cord assembles border astrocyte territories and a fibrotic core with perivascular/meningeal fibroblasts and extracellular matrix, forming a lesion compartment similar in principle to brain scars yet anatomically constrained by spinal architecture [11]. These processes intersect with systemic responses, neuropathic pain pathways, and autonomic dysregulation.

### 3.4. Systemic and Metabolic Causes of Acute CNS Dysfunction

Beyond focal or mechanical lesions, systemic crises such as sepsis, hepatic failure, or metabolic collapse can trigger acute brain dysfunction. These events produce a secondary encephalopathy dominated by endothelial dysfunction, barrier leakage, and cytokine-driven neuroinflammation rather than direct necrosis [15,19]. Experimental sepsis models reproduce the clinical phenotype of fluctuating arousal and delayed cognition through BBB leakage, astrocytic swelling, and microglial activation [20]. Similar cascades appear in hypoxia, hypoglycemia, and hepatic or toxic encephalopathies [21]. Despite diverse triggers, these conditions converge with focal injuries on energy failure, excitotoxicity, inflammation, and maladaptive plasticity, explaining why survivors of sepsis or metabolic coma often experience long-term cognitive and affective deficits comparable to those after stroke or TBI. Current management remains largely supportive, focused on hemodynamic stabilization, infection control, and mitigation of metabolic derangements.

### 3.5. Cross-Cutting Mechanisms and Therapeutic Objectives

Across vascular, traumatic, spinal, and systemic injuries, several intertwined mechanisms determine outcome. Excitotoxic ionic failure is almost universal: as ATP stores collapse, glutamate accumulates and NMDA/AMPA receptors flood neurons with Ca^2+^, initiating protease activation and mitochondrial dysfunction. Parallel neurovascular-unit breakdown exposes the brain to edema and leukocyte infiltration; endothelial tight-junction loss and increased transcytosis compromise the barrier, while pericyte stress and astrocyte detachment further weaken microvascular stability [11]. The resulting leakage amplifies inflammation, which in turn exacerbates oxidative and metabolic stress [4]. Inflammatory amplification follows within hours: activated microglia and infiltrating monocytes release cytokines, chemokines, and reactive oxygen species that can extend cell loss yet are indispensable for debris clearance and repair [17]. In ICH and systemic inflammation, heme and cytokine storms add further toxicity. Over subsequent days, astrocytes and fibroblasts remodel the lesion into spatially distinct domains: a glial border that isolates viable tissue and a fibrotic core that provides structural stability but restricts axonal regrowth [10]. Paralleling glial responses take place in diffuse systemic encephalopathies.

Together, these processes form a continuum that spans etiologies: excitotoxic and inflammatory stresses initiate damage, barrier failure amplifies it, and glial–stromal remodeling seals it off. Understanding this shared logic provides the conceptual foundation for targeting mechanisms, such as neuromodulatory systems of arousal and autonomic regulation that operate across injury types and time scales.

### 3.6. Therapeutic Objectives Across Time: What Current Care Tries to Achieve

**Acute (minutes–hours).** The goal is to limit primary and imminent secondary injury. In ischemic stroke, this means rapid reperfusion of the penumbra by thrombolysis or thrombectomy. In ICH, it entails limiting hematoma expansion through prompt blood-pressure control and reversal of anticoagulation. After cardiac arrest, physiology-directed post-resuscitation care (i.e., stabilizing oxygenation, perfusion, and temperature) aims to reduce global ischemic damage [15]. In systemic crises such as sepsis or metabolic failure, early hemodynamic and metabolic stabilization and infection control are crucial to prevent further hypoperfusion and inflammatory injury [19]. Across etiologies, the intent is to rescue vulnerable tissue, stabilize systemic physiology, and avoid iatrogenic harm during this labile phase.

**Subacute (days).** Priorities shift toward containing secondary cascades: managing edema, preventing hemorrhagic transformation, and controlling seizures or delirium. Barrier and microvascular stabilization become central, alongside calibrated modulation of inflammation, suppressing excess cytokine activity without blocking necessary repair. In systemic insults, maintaining perfusion, oxygenation, and glycemic balance prevents additional neural injury. Early specialist-unit protocols and rehabilitation principles already begin to influence trajectory. Approaches such as minimally invasive hematoma evacuation, targeted blood pressure management, or structured post-sepsis bundles exemplify the effort to translate mechanistic insight into functional gains.

**Chronic (weeks–months and beyond).** Long-term care extends from survival to functional independence, cognition, mood, fatigue, and pain control. Persistent deficits after stroke, trauma, or sepsis often reflect incomplete resolution of secondary cascades rather than residual lesion size. Despite progress in acute interventions, effective strategies to restore arousal stability, motivation, and adaptive plasticity remain scarce. These gaps motivate exploration of neuromodulatory approaches capable of enhancing wakefulness, drive, and circuit reorganization during rehabilitation.

Why does this scaffold matter? This time-ordered framework defines the windows of biological opportunity where therapies targeting arousal, inflammation, and barrier integrity might act, while clarifying the constraints of each condition: reperfusion biology in ischemia, mass effect in hemorrhage, diffuse metabolic–vascular instability after sepsis or cardiac arrest, and compartmentalized scarring in TBI or SCI. The next sections build upon this scaffold to (i) outline orexin biology relevant to these processes, (ii) describe practical means to boost orexinergic tone, (iii) review experimental and clinical evidence, and (iv) discuss a translational outlook that is timing-aware and mechanism-linked.

## 4. The Orexin/Hypocretin System in Brief

### 4.1. Cellular Mechanisms and Receptor Signaling

Orexin-A (hypocretin-1) and orexin-B (hypocretin-2) are neuropeptides encoded by the *HCRT* gene and produced by a small but widely projecting population of neurons in the lateral, perifornical, and dorsomedial hypothalamus [22,23]. Despite numbering only thousands of cells, their axons innervate virtually every brain region, including cortex, thalamus, brainstem, and spinal cord, forming an integrative hub that links metabolic state and external conditions to arousal and behavior.

Two G-protein-coupled receptors mediate orexin signaling. OX_1_ binds preferentially to orexin-A and couples mainly to Gq/11, activating phospholipase C, inositol-trisphosphate (IP_3_) production, and intracellular Ca^2+^ mobilization. OX_2_ binds both peptides with comparable affinity and can signal through Gq/11 and Gi/o, enabling mixed excitatory and modulatory actions [7,24]. Receptor activation closes K^+^ leak channels and opens non-selective cation channels, producing slow depolarizations and sustained firing in target neurons [25].

Downstream cascades couple to CaMKII and ERK/MAPK pathways that support long-lasting potentiation and gene transcription, whereas AMPK–mTOR signaling adjusts firing rate to cellular energy status [25]. Through these interactions, the orexin system helps tune neuronal excitability under fluctuating energy conditions. By integrating energy-balance signals with arousal circuitry, orexins thereby support sustained network activation and protect against synaptic fatigue during prolonged wakefulness [26]. This combination of excitatory and metabolic effects explains why orexin activity stabilizes behavioral states over minutes to hours rather than milliseconds.

### 4.2. State Control and Homeostatic Integration

Orexin neurons operate as sentinels of vigilance and internal balance. They fire tonically during wakefulness, particularly during active exploration or stress, and fall silent in sleep [6]. Their projections innervate every component of the ascending arousal system (e.g., the locus coeruleus (LC), dorsal raphe nucleus (DRN), tuberomammillary nucleus, laterodorsal/pedunculopontine tegmentum, and basal forebrain) where they excite monoaminergic and cholinergic neurons to maintain cortical activation [27]. Degeneration of this network produces the pathognomonic loss of wake stability seen in narcolepsy type 1 [28]. Conversely, selective OX_2_ agonists such as danavorexton and ALKS-2680 sustain alertness without the tolerance typical of psychostimulants [24].

Orexin neurons also integrate autonomic and metabolic cues. They are activated by fasting, hypoglycemia, and hypercapnia, and inhibited by glucose and leptin [29,30]. Projections to the paraventricular nucleus and rostral ventrolateral medulla drive sympathetic outflow, increasing blood pressure, thermogenesis, and respiratory rate [31]. Through AMPK–mTOR coupling, their excitability reflects intracellular ATP levels, ensuring that behavioral activation occurs when energy availability permits [32].

This orchestration makes the orexin network a physiological bridge between homeostasis and action: it estimates energetic need, mobilizes autonomic effectors, and maintains wakefulness long enough for adaptive behavior to occur. In the context of CNS injury, when energy failure, disordered sleep, and autonomic instability are common, this capacity to synchronize metabolism and arousal becomes especially relevant.

### 4.3. From Drive to Movement: Orexins and the Neural Implementation of Action

The transition from internal drive to noticeable movement depends on linking motivational state to motor command, a process in which orexins play a central role. Orexin neurons project to motor cortex, striatum, red nucleus, superior colliculus, cerebellum, and spinal cord [27,33]. Their activation precedes and facilitates voluntary movements: optogenetic or chemogenetic stimulation elicits locomotion and posture adjustments even in resting animals [34]. Recordings show that orexin cells discharge vigorously just before self-initiated actions, encoding not mere arousal but the intention to move [35].

At the supraspinal level, orexin inputs to the nucleus accumbens and ventral tegmental area potentiate dopaminergic signaling, biasing behavior toward high-effort rewards [5,36]. Within motor cortex and pontine reticular formation, orexins enhance neuronal excitability and synchrony, promoting readiness for complex actions [37]. These influences suggest that orexins provide a motivational “gain” on motor networks, ensuring that energy investment aligns with behavioral goals.

Descending orexinergic projections reach spinal motoneurons and interneurons, where OX_1_ and OX_2_ activation depolarizes cells, augments persistent inward currents, and amplifies reflex output [38]. Functionally, this translates into increased muscle tone and motor responsiveness. Such descending facilitation may be critical for maintaining posture and motor engagement during recovery after injury, when descending monoaminergic tone is blunted. By coupling cortical intention, limbic motivation and spinal excitability, orexins effectively transform internal drive into coordinated movement, a principle directly applicable to post-lesional motor rehabilitation.

### 4.4. System-Wide Neuromodulatory Integration

Orexin neurons exist within a dense, reciprocal web of neuromodulatory circuits that collectively shape global brain states. Through these interactions, orexins act more as the conductor of a multi-system orchestra than as an isolated transmitter.

In the LC, orexins excite noradrenergic neurons via OX_1_-dependent depolarization, promoting sustained firing and enhancing cortical norepinephrine levels [39]. The LC, in turn, provides excitatory input back to the hypothalamus, generating a positive feedback loop that sustains alertness [6]. In the dorsal raphe nucleus, orexin-A increases the activity of serotonergic neurons while simultaneously modulating GABAergic inhibition, producing state-dependent shifts in serotonin tone [40]. During stress or novelty, this coupling promotes resilience and behavioral engagement, but chronic over-activation can yield anxiety-like hyperarousal. In the ventral tegmental area, orexin-A potentiates glutamatergic drive onto dopamine neurons through NMDA-receptor phosphorylation, linking arousal and reward [36]. Consequently, orexinergic release scales with motivational salience and anticipated effort, ensuring that dopaminergic reinforcement matches energetic readiness [41]. Additional projections to the tuberomammillary nucleus and basal forebrain excite histaminergic and cholinergic neurons, further amplifying cortical desynchronization and attention [42].

These multiple reciprocal connections enable orexins to synchronize the brain’s arousal, affective, and motor systems into coherent states suited for the organism’s goals. Noradrenergic and dopaminergic excitation heightens vigilance and initiative, serotonergic and cholinergic pathways refine mood and cognition, and inhibitory feedback from GABAergic and serotonergic neurons provides the damping force that prevents metabolic overload. The result is a flexible yet stable framework for adaptive behavior, a mode of global coordination that is frequently disrupted after CNS injury and that orexinergic modulation may help restore.

## 5. Strategies for Enhancing Orexinergic Tone

Efforts to enhance orexinergic tone have grown substantially in the last two decades. However, despite this expanding interest, progress has been slower than for antagonists. While dual orexin receptor antagonists rapidly reached clinical approval for insomnia [43], strategies to boost orexin signaling have faced pharmacokinetic, safety, and delivery challenges. Yet, a diverse toolbox now exists (summarized in Table 1), spanning from small-molecule agonists in late-stage trials to experimental gene and cell therapies, each offering distinct translational opportunities.

### 5.1. Synthetic Small-Molecule Agonists

The most advanced pharmacological tools are small-molecule agonists, mostly OX_2_-selective given its central role in promoting stable wakefulness [24]. Leading compounds include danavorexton (TAK-925), oveporexton (TAK-861), E2086, and alixorexton (ALKS-2680), developed by Takeda, Eisai, and Alkermes, respectively [44,45,46,47]. These agents reliably promote wakefulness in narcolepsy with acceptable tolerability, though their efficacy and safety profiles outside this indication remain untested. Danavorexton, an intravenous short-acting agonist, and oveporexton, an oral longer-lasting analog, have shown the strongest clinical efficacy and are nearing regulatory approval for narcolepsy type 1 [45]. By contrast, the first oral OX_2_ agonist TAK-994, despite robust wake-promoting efficacy, was discontinued after signals of liver toxicity emerged in phase 2, underscoring that safety profiling of this new drug class is still very much in progress. Collectively, these programs nevertheless provide proof of concept that pharmacological replacement of orexin signaling is feasible in humans.

A newer dual OX_1_/OX_2_ agonist, RTOXA-43, increases wakefulness and sleep–wake consolidation in aged mice [48]. Although still preclinical, dual compounds may more closely mimic native orexin signaling across motivational and arousal domains. Overall, OX_2_-selective agonists currently represent the most translationally ready option, but their long-term safety and organ-specific toxicity profiles remain incompletely defined, while OX_1_ activation continues to be an almost unexplored therapeutic frontier.

### 5.2. Peptide-Based Orexin Replacement

Native orexin-A, with high affinity for both receptors, remains the reference ligand for restoring dual orexinergic tone despite its short half-life and limited brain penetration. Central administration demonstrated early feasibility: intracerebroventricular (icv) or intrathecal delivery robustly increased wakefulness and locomotion in rodents [49,50], but invasiveness, instability, and potential desensitization restrict clinical translation [51,52].

Intranasal delivery provides a less invasive alternative [53,54]. Studies in humans and animal models have documented central effects after intranasal orexin-A administration, including modulation of vigilance, sympathetic tone, and even neuroprotection after acute injury, supporting the plausibility of nose-to-brain delivery [55,56]. In parallel, several paradigms report increases in circulating orexin-A and autonomic activation, indicating that peripheral or systemic mechanisms may also contribute. Moreover, the pharmacokinetic evidence remains heterogeneous: central uptake after intranasal orexin-A is variable, often modest, and strongly influenced by BBB permeability, which is transiently increased after acute injury but restrictive in healthy conditions [57,58]. The precise route of nose-to-brain transport (i.e., trigeminal, perineural, or paracellular) remains unresolved. Rapid clearance [57], variable deposition [58] and suprapharmacological doses in rodents further complicate translation. Nevertheless, in states where vascular and immune barriers are compromised, such as acute stroke, trauma, or sepsis, intranasal delivery may provide a transient therapeutic window in which central access is enhanced. Optimized sprays and mucoadhesive compounds, already successful for intranasal insulin and oxytocin [59,60], could improve reproducibility and bioavailability but remain experimental for orexin peptides.

Carrier systems, including nanoparticles, liposomes, exosomes, and BBB-shuttle conjugates, offer additional ways to protect peptides and improve brain access. Transferrin-targeted liposomes increased orexin-A delivery in rats [61], and similar designs with oxytocin or PACAP38 have shown enhanced stability and CNS penetration [62,63]. Although complex and still experimental, these technologies outline a promising route toward long-acting peptide formulations, once issues of manufacturability, scalability, and regulatory approval can be resolved.

### 5.3. Physiological and Behavioral Modulation

Because orexin neurons integrate metabolic, circadian, and motivational cues, physiological interventions that influence these domains can enhance orexinergic signaling endogenously. Orexin neuronal activity rises during wakefulness and energy demand, and stable light–dark schedules with consolidated sleep preserve this rhythm. In contrast, circadian misalignment or sleep fragmentation dampens orexin expression [6,64]. Acute sleep deprivation transiently increases prepro-orexin mRNA and orexin-A levels in rodents and humans, and pre-injury sleep loss can mitigate subsequent CNS damage in some models [65,66], though this remains a preventive rather than therapeutic strategy.

Physical activity is a robust physiological driver of the orexin system. Exercise increases hypothalamic activation and circulating orexin-A [67], and orexin population activity scales closely with locomotion and energy turnover [68]. Conversely, orexin signaling facilitates voluntary movement even when competing with rewarding alternatives [69]. Although model- and context-dependent, this bidirectional interaction may be relevant for rehabilitation.

Nutritional and thermogenic influences further modulate orexinergic excitability. Fasting, ghrelin, and cold exposure activate orexin neurons, whereas glucose and leptin inhibit them [29,30]. Diets that stabilize glycemia or enhance thermogenesis through higher protein intake or moderate ketogenic composition help preserve basal orexin activity [31]. Branched-chain amino acid (BCAA) supplementation increased orexin neuron activation and restored sustained wakefulness in selected TBI paradigms [70], although generalizability across injury types remains uncertain.

Beyond metabolic regulation, orexin neurons are also responsive to novelty, reward, and social complexity. Enriched or stimulating environments increase orexin gene expression and neuronal activation [5], consistent with increased activity in dopaminergic and limbic circuits. Although causal links remain to be fully established, these observations support the idea that behavioral modulation can complement pharmacological strategies by promoting endogenous orexin activity and circuit responsiveness.

### 5.4. Neuromodulation and Electrical Stimulation

Neuromodulation has emerged as a potential way to influence orexinergic circuits indirectly. Transcranial stimulation enhances alertness and cortical excitability, and preclinical work suggests it can modulate orexin-related markers (e.g., orexin-A levels or OX_1_ expression) in preclinical models, engaging downstream signaling cascades associated with neural recovery and homeostatic regulation [71,72]. Although the specificity of this recruitment remains to be clarified, these findings support a top-down route by which cortical activation may influence hypothalamic orexin pathways.

Peripheral approaches such as vagus-nerve stimulation (VNS), transauricular VNS, and median nerve stimulation (MNS) act primarily through ascending noradrenergic and serotonergic nuclei targeted by orexin projections, and orexinergic involvement has been documented in some rodent paradigms [73]. VNS improves vigilance and autonomic stability in humans, consistent with (but not definitively attributable to) orexinergic recruitment [74]. These modalities therefore offer clinically accessible ways to modulate arousal networks that overlap with orexin systems, even if their orexin specificity is incomplete.

Although invasive, hypothalamic deep-brain stimulation (DBS) has been performed safely in small human studies for refractory metabolic and neurological conditions [75,76]. Its relevance for orexin-targeted therapy remains speculative, as selective activation of orexin neurons in humans has not been demonstrated. Nonetheless, neuromodulation bypasses pharmacokinetic constraints and may complement orexinergic pharmacotherapy by enhancing circuit responsiveness, once individual calibration and longitudinal monitoring are feasible.

### 5.5. Cell and Gene Therapy

Biologically restoring orexin signaling has progressed substantially in the context of narcolepsy type 1, where selective orexin cell loss is the primary pathology. Transplanted orexin-producing neurons can survive, integrate, and alleviate symptoms in mouse models [77], and immortalized orexin cell lines grafted into the dorsal raphe restore motor–arousal synchrony and reduce cataplexy, particularly when paired with chemogenetic activation [78]. Gene-therapy approaches re-expressing prepro-orexin in targeted brain regions similarly restore aspects of wakefulness [79,80].

Such strategies, however, are best suited to conditions defined by primary orexin cell loss. In acquired CNS injuries, orexinergic dysfunction is typically secondary, making full replacement less applicable. Key obstacles, including immune rejection, long-term survival, tumorigenicity, and precise integration, remain unsolved [81,82]. Still, recent progress demonstrates that orexin replacement is biologically feasible and increasingly reliable, with immortalized lines and stem-cell-derived hypothalamic neurons offering scalable sources. While direct use in stroke, trauma, or sepsis is improbable, adapted approaches (e.g., viral tools to boost residual orexin output or circuit-specific interventions) may eventually emerge from this line of work.

Taken together, available approaches span a continuum from molecular precision to systemic physiological modulation. The following section examines how these strategies have performed in experimental and clinical contexts of acute CNS damage.

## 6. Experimental and Clinical Evidence of Orexinergic Modulation in Acute CNS Damage

Acute injury to the CNS disrupts the same homeostatic variables that normally drive orexin neurons, including energy balance, vigilance, and autonomic control. The resulting disruption of arousal stability and metabolic regulation creates a context in which the orexin system becomes both highly vulnerable and potentially reparative. Experimental and clinical observations converge on two consistent findings: orexin signaling is altered soon after brain or spinal insults, and interventions that restore or mimic its activity can modulate multiple secondary processes relevant to recovery.

Thus, reductions in cerebrospinal orexin-A levels and changes in hypothalamic or brainstem expression have been reported across ischemic, traumatic, and systemic conditions, often correlating with impaired alertness, motivation, and autonomic function. Conversely, exogenous orexin-A or pharmacological agonists reinstate wakefulness, stabilize metabolic and cardiovascular parameters, and influence neuroinflammatory tone. Together, these data suggest that the orexin system acts as a sensor and regulator of neural integrity, linking cellular stress to organism-level arousal. Nevertheless, current clinical evidence remains scarce and largely observational, and experimental interventions aimed at enhancing orexinergic signaling are still restricted to rodent models. As such, interpretations should be made with caution, and substantial translational work remains before definitive clinical conclusions can be drawn.

In the following sections, experimental and clinical evidence is examined exhaustively by pathological origin, emphasizing mechanistic insights and the temporal windows in which orexin modulation exerts its effects. The relevant literature in this regard is compiled in Table 2 and Table 3.

### 6.1. Cerebrovascular and Global Ischemic Injuries

#### 6.1.1. Orexin Alterations After Ischemic or Hemorrhagic Events

Cerebrovascular insults rapidly disturb hypothalamic and brainstem circuits that sustain orexinergic tone. Across ischemic, hemorrhagic, and global ischemic models, early reductions in orexin-A levels mirror the combined impact of energy failure, oxidative stress, and autonomic dysregulation.

In ischemic stroke, both clinical and experimental data consistently show an acute depression of orexin signaling. Patients exhibit decreased CSF orexin-A within the first 48–72 h, inversely correlating with infarct volume and neurological severity [84,86]. Some cohorts report that higher serum orexin-A during the subacute period predicts faster neurological improvement and better sleep–wake regulation, although in certain cases excessive orexin-A has been linked to post-stroke insomnia [85]. The magnitude and temporal dynamics of orexin suppression vary between studies, however, indicating that orexin depletion is an early but not uniformly sustained feature of ischemic injury. Rodent models of middle-cerebral-artery occlusion (MCAO) confirm this temporal profile, with hypothalamic prepro-orexin mRNA and peptide content declining within the first 6–12 h and recovering over the subsequent days [99,101,107,109,160]. Up-regulation of OX_1_ expression within the lesion core but not OX_2_ [97,98] suggests a stress-induced receptor shift accompanying this early depression.

A comparable pattern is observed in hemorrhagic conditions. In ICH, clinical studies describe a marked acute reduction in CSF and plasma orexin-A, consistent with hypothalamic suppression proportional to hemorrhage severity [87,90,109]. During early recovery, some cohorts report a rebound or overshoot in circulating orexin-A, possibly reflecting autonomic and endocrine stress responses. After SAH, CSF orexin-A follows a biphasic course, with an initial depression during coma and a subsequent transient rise associated with vasospasm and autonomic instability [88,89,90]. Across cohorts, however, although orexin-A frequently correlates with consciousness level and acute neurological impairment, these associations remain variable, with several studies reporting weak or absent relationships with hematoma volume or outcome scores. Preclinical ICH models parallel the clinical course, showing reduced hypothalamic and cortical orexin-A immunoreactivity during the first 24–48 h, followed by progressive recovery [111], though temporal trajectories in animal models tend to be more uniform.

Global ischemic conditions show analogous dynamics. Although clinical data on orexin levels are lacking, studies in rats subjected to asphyxial cardiac arrest show a sharp fall in CSF orexin-A during the post-arrest coma, followed by progressive recovery over 24–72 h that parallels improvements in electroencephalographic (EEG) reactivity and neurological scores [113,114,115,116,118,119,120]. These trajectories reinforce the notion that restoration of orexin tone accompanies the re-emergence of arousal and autonomic stability.

In summary, whether the insult is ischemic (focal or global) or hemorrhagic, orexin-A emerges as a remarkably sensitive barometer of metabolic stress and recovery. Its sharp early fall marks the moment when arousal circuits shut down and neuronal activity is at its lowest. As perfusion improves and systemic physiology stabilizes, orexin levels gradually rise again, tracking the return of cortical responsiveness. Although each condition shows its own temporal signature (progressive recovery in ischemia and more biphasic patterns in hemorrhage) the core principle is the same: changes in orexinergic tone closely follow the transition from acute energetic failure to systemic stabilization.

#### 6.1.2. Experimental Modulation of Orexin Signaling

**Acute-phase interventions.** Administration of orexin during the early acute window mitigates ischemic and hemorrhagic injury in multiple preclinical models. In MCAO, icv orexin-A delivered within 1–3 h reduces infarct volume, improves neurological scores, and dampens apoptotic and inflammatory signaling through ERK, Akt, NF-κB, and MAPK pathways [101,102,105,107,160]. Across studies, these effects appear largely OX_1_R-dependent, consistent with the receptor shifts observed in ischemic tissue [100,111]. In models of hypoxia–ischemia, orexin-A and orexin-B similarly promote cell survival by reducing oxidative stress and engaging pro-survival ERK/Akt cascades [122,123,124]. In ICH, early orexin-A administration via icv or intranasal routes reduces perihematomal edema, limits BBB disruption, and suppresses pro-inflammatory cytokines such as IL-1β and TNF-α [87,111,112]. In this context, orexin-mediated activation of CaMKKβ–AMPK signaling provides a mechanistic link to cellular energy sensing and metabolic stress responses [111]. In SAH, delivery during the vasospasm-prone period improves microvascular reactivity and corresponds with improved arousal scores [88,89]. In global ischemia and post-cardiac-arrest models, both icv and intranasal orexin-A promote faster EEG recovery, stabilize autonomic output, and shorten coma duration [115,116,117,118].

Indirectly enhancing endogenous orexin tone also provides acute benefits. Sleep-deprivation paradigms applied prior to stroke likewise act as preconditioning beneficial stimuli, with transient endogenous orexin elevation reducing infarct size and pro-inflammatory and cell-cycle gene expression [108,161]. Analogously, caloric restriction applied overnight before cardiac arrest improved neurological outcomes and reduced neurodegeneration while modulating systemic metabolism, lowering stress-induced hyperglycemia and elevating ketone bodies, despite no changes in BDNF or SIRT1 expression [121]. Even interventions not designed to target orexin directly may engage this pathway: intravenous parecoxib, a COX-2 inhibitor, increases hypothalamic orexin-A concentration at 72 h post-MCAO, coinciding with reduced damage and improved behavioral scores [110].

Overall, acute-phase evidence across ischemic, hemorrhagic, and global ischemic models demonstrates a convergent early benefit of enhancing orexinergic tone, particularly in suppressing inflammation, reducing oxidative stress, supporting perfusion, and accelerating arousal recovery. Replication is strongest for arousal-related effects, while molecular and metabolic endpoints remain less systematically characterized. Collectively, these data suggest that appropriately timed enhancement of orexinergic signaling can help re-establish arousal and support systemic metabolic recovery following acute cerebrovascular or global ischemic injury. At the same time, broader orexin–autonomic research cautions that strong orexin drive increases sympathetic outflow and cardiovascular tone, so excessive or poorly timed stimulation may elevate tachycardia and hemodynamic load [100,107,119,162].

**Subacute-phase interventions.** During the subacute period, interventions that sustain or restore orexinergic signaling continue to support functional recovery: orexin overexpression improves neurological scores, restores sleep–wake structure, and reduces inflammation across days 1 to 10 after MCAO [109]. In hemorrhagic models, exogenous orexin-A reduces edema and neuronal damage and improves behavioral outcomes across subacute time points, with effects extending to 28 days in some protocols [111,112]. In global ischemia/post-cardiac arrest, improvements in neurological indices persist beyond the immediate hours into several days when orexin-A is delivered early, indicating that acute administration can exert carry-over benefits into the subacute window [115].

Preconditioning strategies also retain protective effects in this window. Sleep deprivation prior to ischemia continues to increase endogenous orexin-A, reducing infarct size and modifying inflammatory and cell-cycle gene programs days after the insult, with concurrent changes in rapid eye movement (REM) sleep architecture [108,161].

Altogether, subacute-phase data support orexinergic interventions as facilitators of network re-engagement and behavioral recovery, particularly in settings where interventions (genetic or pharmacologic) were sustained or where endogenous tone was boosted [108,109,111,112,115,161]. During this window, partial restoration of sleep–wake architecture and autonomic balance, both strongly modulated by orexin, likely contributes to improved arousal and motor readiness, although mechanistic resolution remains limited.

**Chronic and delayed interventions.** Chronic-phase evidence is more limited and uneven across conditions. In ICH models, benefits of orexin-A on neurological function, brain water content, and tissue preservation are reported through 2–4 weeks post-injury [111,112]. For ischemic stroke and global ischemia, most datasets concentrate on acute and early subacute endpoints (hours to ~10 days), so claims beyond that window remain provisional.

Mechanistically, sustained normalization of sleep–wake structure and autonomic balance provides a plausible route for longer-term gains, yet controlled chronic-phase studies and clinical trials are still lacking. Although direct orexin agonists have not yet been trialed clinically after stroke or hypoxic encephalopathy, their pharmacological profile suggests potential utility for restoring vigilance, metabolic regulation, and motor engagement during rehabilitation [24]. Indeed, behavioral or pharmacological strategies that indirectly raise orexin tone, including exercise or structured wake therapy, ameliorate daytime somnolence and improve drive [5], hinting at potential relevance for rehabilitation paradigms after brain injury.

Overall, current animal data point to a shift in therapeutic emphasis from early cytoprotection to circuit and behavioral support in later phases, with the most robust chronic-interval evidence currently coming from ICH models [111,112]. In any case, chronic-phase orexin modulation remains sparsely explored and requires more systematic investigation.

### 6.2. Traumatic Brain and Spinal Cord Injury

#### 6.2.1. Orexin Alterations After Mechanical Trauma

Mechanical trauma to the brain or spinal cord perturbs hypothalamic–brainstem networks that couple arousal, autonomic output, and metabolic control. In traumatic brain injury (TBI), clinical studies consistently show reduced CSF orexin-A during the acute phase, with concentrations remaining low at 6 months post-injury and correlating with post-traumatic hypersomnia [91,92]. Post-mortem analysis shows reduced orexin immunoreactivity, consistent with functional suppression or structural loss of lateral-hypothalamic neurons [93]. Prospective clinical data further support that low CSF orexin-A in comatose TBI or stroke patients predict poorer neurological recovery and higher mortality at 7–14 days, independent of baseline GCS score [94]. These observations suggest that traumatic mechanical forces can induce sustained orexinergic disruption detectable across acute and chronic post-injury intervals.

Preclinical fluid-percussion and controlled-cortical-impact models parallel this phenotype. Mice and rats show decreased *Hcrt* mRNA and peptide content within 24 h, with partial recovery over the following two weeks [70,126,127,128,129,130,141]. This early suppression is accompanied by dynamic changes in orexin receptor expression: in a controlled cortical impact model, OX_1_ immunoreactivity appears around the injury penumbra within 6 h, peaks at 24 h, and gradually declines thereafter. Initially co-localized with activated microglia, OX_1_ expression later becomes neuronal, suggesting a potential shift from early immune modulation to later synaptic or network remodeling [125].

Persistent orexinergic abnormalities become evident in the subacute and subchronic intervals. TBI mice develop chronic hypersomnolence and fragmented wakefulness associated with reduced hypothalamic activation [70]. Similar sleep–wake instability and attentional deficits are reported during weeks 2–4 post-injury [126,128,129], with abnormal spectral power dynamics and impaired hypothalamic–brainstem coupling during recovery [127,132]. Collectively, these findings suggest that incomplete restoration of orexin tone and associated circuit integrity contributes to fatigue, poor vigilance, and reduced environment engagement, aligning with clinical observations of persistent sleep–wake disturbances.

Structural and circuit-level changes may further underlie these deficits. In repeated mild TBI models, orexin-producing neurons are diminished, and projections to the periaqueductal gray, critical for defensive responses and nociception, are weakened [142]. These anatomical changes coincide with altered nociceptive thresholds and reduced anxiety-like behaviors, indicating dysregulation of the arousal–pain interface.

To date, no study has directly examined orexin alterations after SCI. However, SCI induces several hypothalamic changes that could plausibly affect orexinergic function including metabolic, neuropeptidergic, and autonomic alterations. Reported adaptations include disrupted leptin and adipokine signaling [163], altered hypothalamic POMC and NPY expression [164], and plasticity within lateral-hypothalamic circuits that modulate locomotor recovery [165]. Although indirect, these observations point to a significant knowledge gap and raise the possibility that hypothalamic orexin signaling contributes to post-injury autonomic dysregulation, metabolic imbalance, and motivational deficits in SCI.

#### 6.2.2. Experimental Modulation of Orexin Signaling

**Acute-phase interventions.** In TBI, evidence supporting a beneficial effect of direct orexin replacement within the acute window after trauma is scarcer than in cardiovascular conditions. A recent study reports that exogenous orexin-A administration following cortical injury attenuates neuroinflammation, ferroptosis and oxidative damage, leading to preserved tissue structure and improved neurological scores [131]. But more effort is required to replicate this data.

In contrast, a broad set of neuromodulation strategies applied within the first 24–72 h after trauma have been shown to mitigate secondary injury and accelerate early neurological recovery through the activation of endogenous orexinergic circuits. These include VNS, trigeminal nerve stimulation (TNS), and MNS, all of which increase hypothalamic orexin activity and ameliorate early neurological deficits via OX_1_-dependent pathways [73,134,135,138,139]. Similarly, DBS of the lateral hypothalamus delivered during the first 12 h post-injury enhances consciousness and increases both orexin-A and OX_1_ expression. These effects are abolished by OX_1_ antagonism, confirming orexinergic involvement [139]. Another promising approach is low-intensity focused ultrasound stimulation (LIFUS), applied at 3 days post-TBI. LIFUS produced parallel protective effects, reducing edema, inflammasome activation, and oxidative stress while improving behavioral outcomes [140]. Although the exact contribution of orexin in LIFUS benefits has not been directly isolated, LIFUS increased both orexin-A and OX_1_ expression.

Taken together, these acute-phase interventions suggest that boosting orexinergic output, either directly or via upstream neuromodulatory pathways, can reduce neuroinflammation, limit early cell death, and support arousal recovery. However, most studies are preclinical and vary in stimulation parameters, making it difficult to generalize precise dose–response relationships or to define shared mechanisms across modalities.

**Subacute-phase interventions.** At later time points, orexin-related manipulations continue to influence recovery. Thus, interventions that support orexin output tend to stabilize arousal, whereas strategies that bias the system downward can exacerbate sleep–wake instability. In a fluid-percussion injury model, BCAA supplementation initiated 2 days post-injury and maintained for a month, improved wakefulness and partially restored orexin-neuron activation, reversing TBI-induced increases in non-rapid eye movement (NREM) sleep and wake fragmentation [70]. In contrast, sustained suppression of orexin signaling yields an opposing pattern. Daily administration of the dual orexin receptor antagonist DORA-22 for 30 days following controlled cortical impact reduced post-traumatic seizures and enhanced GABAergic inhibition [133]. However, this intervention increased overall sleep pressure and homeostatic sleep drive, worsening hypersomnolence. Similarly, DORA administration across a two-month period induces persistent sleepiness without correcting the EEG abnormalities produced by TBI [132]. This dual profile indicates that lowering orexin tone may have anticonvulsant benefits but also compromises arousal maintenance, raising the possibility—still untested—that pharmacological increases in orexinergic tone could conversely influence seizure susceptibility in TBI.

Overall, subacute interventions reveal that modulating orexin tone (either upward or downward) can significantly alter recovery dynamics, but the evidence base remains limited and heterogeneous, underscoring the need for targeted temporal studies that align orexin manipulation with the evolving state of arousal circuitry after TBI.

**Chronic-phase interventions.** Paralleling persistent orexin deficits found in humans after TBI, lack of orexinergic tone in chronic stages is associated with worse functional outcomes: in a mouse model, a single DORA administration after controlled cortical impact increased sleep fragmentation and sleepiness and promotes hypersomnolence across multiple timepoints (24 h, 2 weeks, 1 month, and 2 months), reinforcing the sensitivity of chronic recovery to orexinergic balance [132]. Yet chronic orexin-A supplementation does not uniformly confer benefits. In a rat model of repeated mild TBI, icv orexin-A administered from day 5 onward for four weeks exacerbated anxiety-like behavior, impaired motor performance, and altered CSF metabolomic and proteomic profiles, contrasting sharply with modafinil, which improved these same parameters despite acting on arousal systems [143]. These findings suggest that chronic orexinergic stimulation may disrupt homeostatic recovery mechanisms and precipitate maladaptive arousal states.

Collectively, human and animal data indicate that long-term orexinergic dysfunction contributes to chronic hypersomnolence and reduced vigilance after TBI, but also that untimely or excessive replacement may be counterproductive, underscoring the importance of precise temporal matching between intervention and circuit state.

In contrast to TBI, evidence directly examining orexin modulation after SCI is extremely limited. One rodent study using complete transection reported that intrathecal orexin-A administered daily for three days enhanced locomotor recovery and normalized glutamatergic transmission within the first week post-injury, suggesting that orexinergic stimulation can acutely modulate spinal network excitability and metabolic balance [144]. While promising, this represents a single dataset, and orexin-specific contributions remain difficult to isolate. Importantly, a recent groundbreaking study demonstrated that DBS of the lateral hypothalamus markedly improved walking ability and motor coordination in individuals with chronic SCI [166]. Although hypothalamic DBS activates diverse neuronal populations, the re-engagement of descending hypothalamic pathways, which include orexin neurons, is a plausible contributor to these motor improvements [139]. Still, the current SCI evidence remains largely inferential. Very few studies explicitly target orexin, and most observations reflect upstream hypothalamic modulation rather than direct orexinergic interventions. Thus, while reactivating hypothalamic output appears beneficial for motor recovery, defining the specific role of orexin in SCI will require controlled, orexin-focused studies.

### 6.3. Systemic Biological and Toxic Insults

#### 6.3.1. Orexin Alterations During Sepsis and Systemic Metabolic Failure

Systemic inflammatory and toxic insults profoundly disturb the neural systems that sustain arousal and homeostatic balance. The orexin network, situated at the interface of metabolic, immune, and autonomic regulation, appears particularly vulnerable to this disruption. In clinical sepsis, CSF orexin-A concentrations are consistently reduced during the acute phase and gradually normalize as systemic inflammation resolves [95,96]. This recovery parallels improvements in consciousness and autonomic stability, suggesting that orexin suppression reflects a state-dependent functional shutdown rather than irreversible neuronal loss. Similar trajectories have been described in isolated case reports where severe sepsis produced markedly low CSF orexin-A accompanied by BBB leakage, both of which reversed with clinical improvement [95]. Although clinical datasets remain limited, the convergent pattern suggests that orexin-A might behave as a dynamic marker of global physiological stress in sepsis.

Preclinical studies show a similarly rapid and reversible suppression of orexinergic activity. Peripheral immune activation with lipopolysaccharide (LPS) silences hypothalamic orexin neurons within hours, producing dose-dependent reductions in orexin immunoreactivity and prepro-orexin mRNA that partially rebound at later time points [147,148,149]. These changes coincide with reduced wakefulness and increased NREM sleep, closely mirroring the hypoarousal observed in human septic encephalopathy [151,152]. Loss-of-function models reinforce this link: orexin-deficient mice exhibit exaggerated sickness behavior, prolonged inactivity, and higher mortality after LPS, indicating that endogenous orexins normally exert a counter-inflammatory and arousal-supporting role [146,150].

The impact of inflammation on orexin neurons is not uniform across behavioral and circadian contexts. In vivo activity mapping confirms that LPS differentially modulates orexin subpopulations depending on behavioral context and circadian phase, suppressing Fos expression in lateral orexin neurons during exploratory behavior, and blunting activation across both orexin and histaminergic neurons during the dark period [145]. Additional studies reveal transcriptional downregulation of prepro-orexin and decreased orexin-A levels across cortex, hippocampus, and pons, effects that are accentuated in orexin-deficient mice [154]. Together, these findings suggest widespread but reversible orexinergic silencing during systemic inflammation, with circuit-level consequences for arousal and autonomic stability.

Not all systemic insults produce uniform suppression, however. In a non-infectious model of systemic inflammation, rats subjected to acute pancreatitis displayed elevated brain levels of orexin, β-endorphin, and oxytocin as early as 12–24 h post-injury despite minimal cytokine induction or microglial activation [153]. This pattern suggests a potential early neuroendocrine alarm response, in which orexin may be transiently upregulated prior to immune-cell recruitment. Similarly, alcohol-induced coma suppresses hypothalamic orexin activity, while exogenous orexin-A/B accelerates awakening and reduces cortical delta power in an EEG pattern consistent with arousal promotion, partly via histaminergic, noradrenergic, and serotonergic pathways [159].

Taken together, available evidence supports a model in which systemic inflammation, toxicity, and metabolic stress produce rapid and predominantly suppressive effects on orexinergic tone, contributing to hypoarousal, altered autonomic regulation, and disrupted sleep–wake cycles during acute illness. Exceptions such as pancreatitis highlight that orexin responses can be context-dependent and may reflect distinct neuroendocrine adaptations depending on the nature and timing of systemic stress.

#### 6.3.2. Experimental Modulation of Orexin Signaling

**Acute-phase interventions.** During the acute phase of systemic inflammatory or metabolic insults, multiple studies support a time-sensitive, OX_1_- or OX_2_-mediated protective role for orexin-A. In rodent models of endotoxemia induced by LPS, centrally or peripherally administered orexin-A (as it crosses the compromised BBB) improves survival, restores cardiovascular tone and thermoregulation, and reduces both plasma and brain cytokine expression, effects that are dependent on OX_1_ activation and vagal cholinergic signaling [157,158]. In a polymicrobial sepsis model (cecal ligation and puncture, CLP), intranasal orexin-A delivered within 30 min post-insult reduces mortality, cerebral edema, BBB disruption, microglial activation, and cognitive impairment through OX_2_-mediated suppression of the RAS/MAPK cascade [155]. Conversely, delayed central administration at 24–48 h post-CLP fails to confer benefit and may exacerbate lethargy and neuroendocrine suppression [154], underscoring the narrow therapeutic window in which orexinergic support remains adaptive. Chemogenetic activation of orexinergic neurons in CLP mice, as well as pharmacological stimulation with xanomeline, also improved physiological arousal and suppressed cytokine production [156]. These protective effects were reversed by dual orexin receptor blockade, supporting a causal link between orexin activity and host resilience. Beyond infectious or inflammatory challenges, orexin also modulates arousal in toxic-metabolic states as seen in a model of alcohol-induced coma, where icv orexin-A/B shortened the duration of unconsciousness and normalized righting reflexes [159]. In this setting, orexin peptides enhanced cortical excitability and reduced EEG delta-power through coordinated recruitment of histaminergic, noradrenergic, and serotonergic pathways. Collectively, these findings identify orexin-A as a fast-acting neuromodulator that bridges metabolic, immune, and arousal systems: when administered early, it stabilizes autonomic and neuroimmune function, enhances survival, and restores consciousness across diverse acute systemic and metabolic insults.

**Subacute-phase interventions.** In the subacute post-septic phase (24–72 h), the focus shifts from survival to restoration of arousal and cognitive function. Although no studies have initiated orexin-based treatments exclusively during this window, the enduring benefits of early administration suggest that early orexinergic support can shape later recovery trajectories. In humans, CSF orexin-A progressively normalizes over the first 1–3 weeks following sepsis, coinciding with improved consciousness and autonomic tone [95,96]. In rodents, the benefits of early orexin-A dosing persist at 7 days post-CLP, with sustained improvements in cognition and structural integrity [155]. Thus, while direct subacute-phase interventions remain unexplored, existing data imply that orexinergic tone contributes to recovery once systemic inflammation begins to resolve, supporting arousal re-engagement and counteracting prolonged sickness behavior. More systematic studies are needed to establish whether this window could be directly targeted.

**Chronic and delayed interventions.** Persistent orexin deficiency after sepsis or critical illness may contribute to post-intensive-care syndrome, characterized by fatigue, sleep disturbance, and cognitive impairment [19]. However, no chronic-phase interventional studies have been performed. Whether long-lasting alterations in orexinergic tone directly underlie post-ICU fatigue or cognitive slowing remains speculative. Yet the pattern of clinical recovery suggests that orexin neurons are capable of substantial plasticity and rebound once systemic stability is regained, opening the door to longer-term neuromodulation strategies that have yet to be systematically examined.

### 6.4. Mechanistic Convergence Across Injury Types

**Figure 1:**
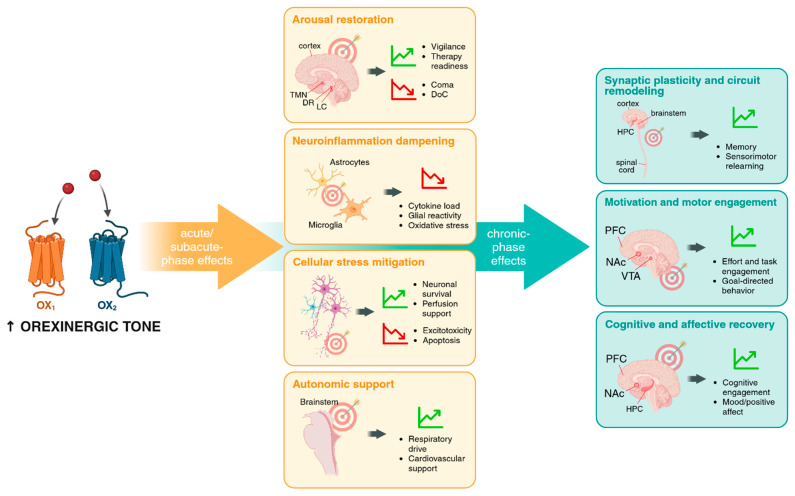
Integrative framework for orexin-mediated mechanisms across phases of recovery after acute CNS injury. Increased orexinergic tone through OX_1_R and OX_2_R engagement is proposed to exert acute- and subacute-phase effects on arousal nuclei, glial populations, vulnerable neurons, and autonomic brainstem centers. These actions may enhance vigilance and therapy readiness, reduce cytokine load, glial reactivity, and oxidative stress, support neuronal survival and perfusion, and stabilize respiratory and cardiovascular output. In later phases, partial recovery or targeted enhancement of orexin signaling may facilitate synaptic plasticity and circuit remodeling, and promote motivation, cognitive engagement, and positive affect. The brain regions illustrated (e.g., LC, DR, TMN, PFC, NAc, VTA, HPC, spinal cord) represent plausible sites where orexinergic modulation may contribute to these processes, based on available experimental evidence across ischemic, traumatic and systemic models. The framework is conceptual and integrates cross-etiology findings rather than depicting a single validated pathway. Green schematic graphs represent increases in the corresponding functions or effects, whereas red graphs indicate their reduction. Abbreviations: LC, locus coeruleus; DR, dorsal raphe; TMN, tuberomammillary nucleus; PFC, prefrontal cortex; NAc, nucleus accumbens; VTA, ventral tegmental area; HPC, hippocampus; DoC, disorders of consciousness; OX_1_/OX_2_, orexin receptor 1/2. Created in BioRender.com. Otero-Lopez, 2026. https://BioRender.com/wvgjzz5.

Acute injuries to the CNS—whether ischemic, traumatic, or systemic—precipitate rapid disruptions in arousal, autonomic regulation, immune activation, and metabolic control. Although each pathology triggers its own cascade, a set of shared mechanistic motifs emerges across models, while important divergences constrain how orexin-targeted interventions might operate. Figure 1 provides an integrative overview of these dynamics, illustrating how orexinergic mechanisms evolve from acute suppression toward circuit and behavioral re-engagement across phases of recovery. Below, we synthesize the convergent patterns, etiology-specific divergences, and temporal transitions, highlighting where evidence is solid, where it is suggestive, and where substantial gaps remain.

#### 6.4.1. Convergent Mechanisms: What Is Consistently Observed?

Across ischemic stroke, ICH, traumatic brain injury, and sepsis, early orexin suppression is one of the most reproducible findings [87,90,99,101,109,113,115,160]. Hypothalamic orexin neurons downshift firing and transcription within hours of injury, paralleling EEG slowing, hypoarousal, and autonomic instability [92,116]. This silencing likely reflects a conserved state-dependent protective down-regulation in the face of metabolic crisis, tissue hypoperfusion, and intense inflammatory signaling. In all etiologies, early restoration of orexin tone, whether via exogenous orexin-A, neuromodulation (e.g., VNS, MNS, DBS), chemogenetics, or endogenous physiological rebound, improves arousal and stabilizes autonomic stability [115,117,134,138,139]. Several models also show reductions in inflammatory cascades and secondary neuronal injury [102,111,112,155,160].

Another area of broad convergence is neuroimmune modulation. Across models, orexinergic enhancement dampens microglial activation, reduces pro-inflammatory cytokines (IL-1β, TNF-α), and limits NF-κB/MAPK signaling [102,117,155,160]. These effects involve OX_1_-mediated inhibition of inflammatory pathways in ischemia and trauma [100,111], whereas some OX_2_-linked effects emerge in hemorrhagic and septic contexts, particularly those related to microvascular tone and autonomic balance [155]. Similarly, re-engagement of ascending arousal systems (LC, dorsal raphe, basal forebrain) during the subacute period is consistently observed when orexin tone rebounds, supporting sleep–wake stabilization and attentional responsiveness [5,109].

Together, these findings define a conserved core: acute orexin suppression accompanies physiological collapse, followed by phase-dependent reactivation that supports neuroimmune stability, arousal recovery, and circuit re-engagement.

#### 6.4.2. Divergent Mechanisms: What Varies Between Etiologies?

Despite these cross-etiology themes, significant differences limit the extent to which a single mechanistic cascade can be generalized.

ICH provides the clearest evidence for direct orexin–bioenergetic coupling, particularly OX_2_-driven activation of CaMKKβ–AMPK, linking orexin to cellular energy sensing and stress–response pathways [111]. This stands in contrast to ischemia, where “metabolic effects” of orexin are usually inferred indirectly (e.g., improved perfusion, reduced oxidative stress) and rarely quantified through direct mitochondrial or ATP measurements. Indeed, neuroimmune benefits are well documented in ischemic stroke, but mechanistic depth varies and many endpoints remain inferred rather than directly measured [106,107,167].

Traumatic brain injury introduces an additional layer of complexity: in this context, not all orexin interventions are beneficial. Chronic orexinergic supplementation may worsen anxiety-like behavior and cognition in repetitive mild TBI, highlighting the risk of maladaptive stimulation during circuit reorganization [143]. DORA reduces seizures but exacerbates hypersomnolence [133]. Conversely, neuromodulatory interventions (VNS, MNS, DBS, LIFUS) robustly improve arousal and attenuate neuroinflammation, largely through OX_1_-dependent mechanisms [134,138,139,140], yet these approaches remain untested in ischemic or septic contexts.

In systemic inflammation, orexin suppression shows marked context- and circadian-dependence. LPS preferentially silences lateral orexin neurons during exploratory behavior and globally blunts orexin–histamine activation in the dark phase, revealing that inflammatory effects on the orexin system depend strongly on behavioral state and intrinsic rhythmicity [145,147,148,151,152]. By contrast, non-infectious systemic insults such as pancreatitis may induce transient orexin elevations rather than suppression [153]. This state dependence entails therapeutic implications, particularly when defining time windows in which exogenous orexin may be most effective.

These divergences emphasize that orexinergic pathways do not respond uniformly to injury, and that therapeutic interventions cannot rely on a single causal narrative.

#### 6.4.3. Temporal Evolution: From Acute Stabilization to Chronic Circuit Dynamics

Across etiologies, orexin-mediated mechanisms evolve through partially distinct temporal phases, consistent with the transitions illustrated in Figure 1.

**Acute phase (hours):** Energetic stress, immune activation, and network disruption dominate. Orexin silencing contributes to coma, autonomic collapse, and reduced respiratory drive [92,116]. Early orexinergic enhancement may stabilize arousal and limit secondary inflammatory injury [112,115,155,160], but excessive stimulation risks sympathetic overload [100,119]. Several findings (e.g., reduced oxidative stress, CaMKKβ–AMPK engagement in ICH, stabilized perfusion in stroke/sepsis) suggest a bioenergetic component [107,111]. Thus, orexinergic silencing might serve a protective, energy-saving function, integrating metabolic cues such as glucose, lactate, and pH via ATP-sensitive K^+^ channels [30,168] to minimize metabolic demand. This remains speculative but mechanistically plausible.

**Subacute phase (days):** As metabolic and hemodynamic conditions stabilize, orexin activity generally begins to recover. The available evidence shows that this rebound often coincides with improvements in barrier integrity [106,119], reduced microglial activation, and more regulated cytokine profiles [102,117,160], consistent with a shift toward a more regulated neuroimmune environment. At the circuit level, partial restoration of orexin tone appears to support re-engagement of ascending arousal pathways, contributing to sleep–wake consolidation and early behavioral activation [109]. Subacute orexin administration improves neurological or autonomic outcomes in several contexts [111,112,155]. Yet, orexin manipulation during this fragile phase can yield divergent outcomes: increasing orexin tone can enhance arousal, whereas orexin blockade may reduce post-traumatic seizures at the cost of exacerbated sleepiness and impaired vigilance [133]. These bidirectional outcomes underline that, in the subacute period, the effects of orexin modulation are highly dependent on timing and physiological state, and cannot be generalized across injuries.

**Chronic phase (weeks to months):** Long after injury, residual orexin dysregulation is thought to contribute to symptoms such as fatigue, apathy, sleep fragmentation, and dysautonomia, well described in survivors of TBI and critical illness [92,95,129], and less consistently in stroke or SCI. These features do not arise exclusively from orexin pathways, but persistent hypo-orexinergic states provide a plausible contributor to chronic hypoarousal and reduced engagement. Conversely, excessive or poorly timed orexin enhancement may be counterproductive. In repetitive mild TBI, chronic orexin-A administration worsens anxiety-like behavior and cognitive performance [143], illustrating that orexin signaling can be maladaptive when pushed beyond physiological needs, particularly during long-term circuit reorganization. Over this interval, interventions that gently support orexinergic tone, whether through behavioral activation, environmental enrichment, or selective pharmacology, may help reinforce motivation, circadian alignment, and cognitive engagement [5]. This phase highlights the need for fine-tuned, receptor-selective interventions temporally aligned with circadian and rehabilitative patterns, as the distinction between therapeutic and disruptive stimulation becomes increasingly narrow.

#### 6.4.4. Conceptual Boundaries and Remaining Uncertainties

Although the integrated framework above captures broad patterns, several key uncertainties remain. First, the bioenergetic dimension, while conceptually compelling, is only partially supported by existing evidence. Explicit links to AMPK signaling, oxidative stress reduction, and microvascular perfusion appear in selected models [111], but systematic evaluation of ATP production, mitochondrial dynamics, or metabolic flux after orexin interventions is scarce. Second, strong context- and state-dependence complicates translation. Orexin responses differ across behavioral states, circadian phases, and injury types, and both clinical and experimental data highlight variability that is not yet mechanistically resolved [145,151,152]. Third, human evidence, particularly longitudinal, remains limited, with inconsistent correlations between orexin levels and outcome measures in certain stroke and hemorrhage cohorts [85,87]. Finally, interventions that increase orexinergic tone are not uniformly beneficial; chronic orexin-A supplementation can worsen behavioral outcomes in repetitive TBI [143], and poorly timed orexin delivery in sepsis may increase metabolic demand before hemodynamic stabilization [154].

These uncertainties reinforce a central point: orexin should not be conceptualized as a single “pro-recovery” pathway but as a state-dependent homeostatic integrator whose therapeutic utility depends on precise timing, receptor targeting, and alignment with evolving circuit conditions. This perspective anchors the translational framework developed in the next section.

## 7. Translational Opportunities, Challenges and Perspectives

**Figure 2:**
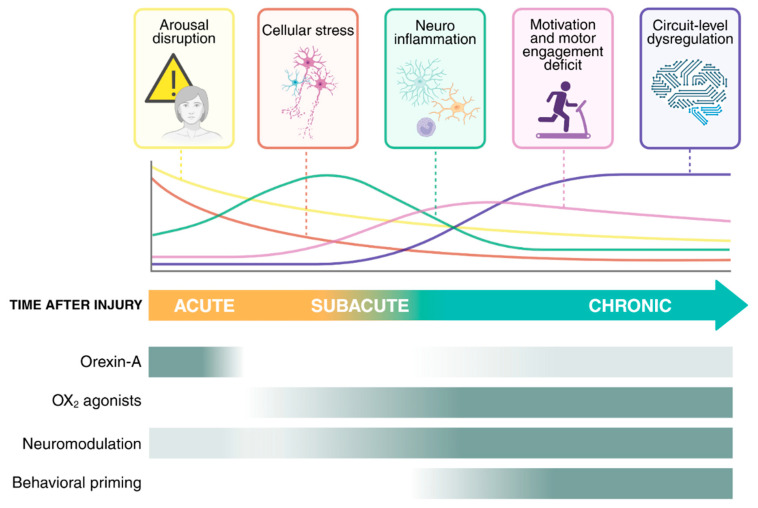
Phase-aligned framework for orexin-based interventions after acute CNS injury. Conceptual overview illustrating how major post-injury processes (arousal, metabolic/bioenergetic stabilization, neuroinflammation, and circuit/motivational engagement) evolve over time and how different orexin-enhancing strategies may theoretically align with these temporal transitions. Proposed orexin-enhancing strategies may differentially support recovery depending on timing: early orexin-A for arousal stabilization, OX_2_ agonists for subacute and chronic hypoarousal, neuromodulatory approaches (e.g., VNS, MNS, and DBS) across phases, and behavioral/metabolic priming interventions (graded physical activity, structured engagement, and nutritional support) primarily in long-term rehabilitation. Lighter shading indicates greater uncertainty about timing or therapeutic benefit. This framework is conceptual and does not imply clinical efficacy of any specific intervention, which remains to be established. Created in BioRender.com. Otero-Lopez, 2026. https://BioRender.com/hez4df5.

Therapeutic modulation of the orexin system holds conceptual potential across acute CNS injuries, but translation requires precision. Because orexin neurons operate at the intersection of metabolic state, neuroimmune reactivity, autonomic control, and motivational drive, their responsiveness varies markedly across the evolution of injury and across the circadian cycle. As a result, orexin-based therapies cannot be applied uniformly: their potential benefits and risks are tightly contingent on the timing, method, and intensity of stimulation, as well as the physiological substrates that remain intact at each stage. Figure 2 provides an overview of how the dominant pathophysiological processes evolve after injury and proposes a conceptual alignment of orexinergic interventions with these transitions.

### 7.1. Temporal Precision: From Injury Phase to Circadian Alignment

Translating orexin biology into therapy requires precision not only in *when* after injury treatment is initiated, but also in *when within the day* it is delivered. The first dimension (phase-specific timing) reflects the evolving biology of injury, from metabolic crisis to recovery, as detailed previously. The second dimension (the circadian phase) acknowledges that orexin neurons are integral components of the sleep–wake oscillator and that their activation must respect intrinsic rhythmicity to remain beneficial.

In the acute phase, when perfusion failure, metabolic collapse, and inflammatory surges dominate, orexinergic stimulation must remain modest, time-limited, and tightly controlled to avoid sympathetic overload and increased metabolic demand. Short-acting orexin-A is mechanistically suited for this narrow window: it engages both receptors, activates cytoprotective and anti-inflammatory cascades, and rapidly restores arousal, but its cardiovascular load and short half-life argue against sustained use. By contrast, longer-acting OX_2_ agonists are less attractive as an early intervention: they do not reproduce OX_1_-dependent cytoprotection and arousal-promoting effects, and prolonged stimulation during hemodynamic instability could increase metabolic strain.

As the system moves into the subacute phase, and metabolic and hemodynamic stability begin to return, therapeutic priorities shift from crisis containment toward the cautious re-engagement of arousal networks, consolidation of early sleep–wake structure, and initiation of rehabilitation. Circadian rhythmicity gradually re-emerges, and orexin activity should peak again in the light–active period. Interventions that synchronize with this rhythm, administered in the morning or early active phase, can amplify arousal and motor engagement while preserving sleep-dependent consolidation at night [5,24]. Short-acting OX_2_ agonists with 1–3 h half-lives become attractive tools to stabilize daytime wakefulness and counter pathological sleep pressure, whereas peptide orexin-A is less advantageous beyond brief, session-specific boosts. Neuromodulatory approaches, including VNS, MNS and DBS of orexin-responsive targets, may also become more effective as ascending networks regain responsiveness. Behavioral or metabolic priming, such as graded physical activity, environmental enrichment, or BCAA supplementation in selected TBI paradigms, can begin to stimulate endogenous orexin firing, though evidence remains preliminary and largely restricted to specific models.

In the chronic phase, long after the initial insult, the dominant barriers to recovery are no longer metabolic fragility or inflammatory cascades but persistent hypoarousal, fatigue, apathy, cognitive slowing, and dysautonomia. Orexin neurons are typically structurally preserved but functionally dysregulated, making them suitable targets for sustained, circadian-aligned interventions. OX_2_-selective agonists are particularly well suited here: they reliably enhance vigilance with relatively low sympathetic load and can be delivered orally early in the morning to support engagement in daily activities and rehabilitation [8,24]. Short-acting orexin-A may in principle serve as a pre-rehabilitation primer to transiently enhance motivation or task engagement, though chronic peptide dosing should be approached cautiously given maladaptive behavioral effects reported in repetitive mild TBI. In this stabilized physiological landscape, neuromodulation and behavioral priming become central: structured exercise, enriched environments, cognitive–motor training, and light-based circadian interventions naturally activate orexin neurons and other arousal circuits, and pharmacological orexin enhancement may be most effective when embedded within these multimodal strategies [5,34].

Systemic inflammation introduces an additional layer of temporal complexity. Experimental data show that LPS suppresses orexin neurons in a highly state- and circadian-dependent manner (i.e., more strongly during exploratory behavior and during the dark period) whereas non-infectious systemic insults such as pancreatitis may transiently increase orexin rather than suppress it. This variability suggests that certain inflammatory states may constitute optimal windows for exogenous orexinergic rescue, whereas others may yield weaker or less predictable responses.

Ultimately, orexin therapy should be understood as chronobiological modulation rather than static replacement. Its effectiveness will depend on delivering the right magnitude of stimulation, at the right injury stage, and at the right time of day to cooperate with endogenous arousal cycles and rehabilitation schedules. This dual precision (i.e., phase-sensitive and circadian-sensitive) represents the conceptual cornerstone of orexin translation.

### 7.2. Delivery Routes and Formulation Strategies

Therapeutic translation depends on reaching central targets efficiently and safely, which in turn reflects whether the compound is a peptide or a small molecule. Peptide-based orexin analogs are rapidly degraded and cross the BBB poorly, so systemic delivery is ineffective. Intranasal administration is the most practical route, enabling direct olfactory and trigeminal transport and measurable hypothalamic activation within minutes [56]. Formulations using nanoparticles or lipid carriers further improve uptake while limiting peripheral exposure [54,63]. These approaches are best suited to experimental or short-term use during the acute and subacute phases, particularly when BBB permeability is transiently increased and when brief modulation of hypothalamic tone can influence arousal trajectories.

Small-molecule orexin receptor agonists overcome these limitations. Drugs such as danavorexton and its oral derivatives display high receptor selectivity, central penetration, and favorable pharmacokinetics [8]. Intravenous danavorexton produces rapid, controllable arousal in humans, while oral analogs offer convenient dosing for longer-term therapy, though safety optimization continues. Their flexibility enables parenteral use for hypoarousal in clinical settings and oral use during rehabilitation to help sustain motivation and energy.

Future formulations may combine fast intranasal peptides with long-acting oral or subcutaneous small molecules, or incorporate circadian-timed release to align orexin exposure with natural wakefulness peaks. Collectively, these delivery options turn orexin modulation into a realistic pharmacological strategy adaptable to both acute stabilization and chronic recovery.

### 7.3. Receptor Selectivity and Subtype Balance

Balancing activity across orexin receptor subtypes is crucial for both efficacy and safety. OX_1_ and OX_2_ share structural homology but mediate distinct physiological domains: OX_1_ drives rapid excitatory, arousal-promoting and immune-modulating responses, and is responsible for much of orexin’s cytoprotective and anti-apoptotic signaling after ischemia or trauma, whereas OX_2_ stabilizes wakefulness and autonomic tone [7,169].

Pharmacological development, however, remains asymmetric. Most clinical progress has focused on OX_2_-selective agonists, which reliably enhance vigilance with minimal sympathetic load [8]. In contrast, no selective OX_1_ agonists and very few true dual agonists are currently available, leaving a therapeutic gap for conditions where OX_1_ signaling predominates. Developing safe small-molecule OX_1_ agonists and balanced dual agonists therefore represents a priority for translational neuroscience. Such compounds could reproduce the full spectrum of orexin’s benefits while allowing phase-specific receptor targeting to minimize adverse autonomic effects, all without the pharmacokinetic limitations inherent to orexin peptides.

### 7.4. Combination and Multimodal Approaches

Because orexins regulate metabolism, arousal, and motivation, its therapeutic effects will likely be maximized through complementary interventions rather than monotherapy. Orexin neurons closely interact with serotonin, noradrenaline, dopamine, and histamine systems [5,7], suggesting that modest co-activation of these circuits could enhance recovery while avoiding excessive sympathetic drive. For instance, serotonergic or noradrenergic agents may stabilize mood and cortical excitability in patients treated with OX_2_ agonists, partially compensating for the current lack of selective OX_1_ agonists.

Behavioral and neuromodulatory combinations hold equal promise. Physical training, environmental enrichment, and cognitive engagement naturally stimulate orexinergic firing and promote plasticity; short-acting orexin agonists given before rehabilitation sessions could therefore prime motivation and learning, particularly in patients with marked daytime somnolence or apathy [34]. Likewise, VNS or transcranial stimulation can boost endogenous orexin release and autonomic stability, potentially amplifying pharmacological effects [134]. Metabolic interventions such as BCAA supplementation in selected TBI models illustrate how nutritional strategies may converge on orexin neurons to restore wakefulness and normalize sleep architecture, although translation of these findings to broader clinical populations will require careful testing.

These integrative approaches position orexins not as stand-alone drug targets but as a hub within a therapeutic network, aligning pharmacological, behavioral, and physiological interventions toward shared recovery goals. Systematic testing of such multimodal paradigms, with careful attention to timing, dose, and patient heterogeneity, will be essential to define optimal combinations and maximize functional outcomes.

### 7.5. Safety Considerations and Potential Risks

Because orexins modulate multiple physiological systems, safety evaluation must extend beyond the CNS. The most consistent preclinical concern is sympathetic overactivation, leading to hypertension, tachycardia, or metabolic strain [8,24]. Beyond autonomic load, excessive or mistimed orexinergic stimulation could theoretically exacerbate hyperarousal-related symptoms such as sleep fragmentation or agitation, particularly in patients with brain injury or systemic illness whose arousal circuits are already unstable. Thus, excessive OX_1_ stimulation can precipitate anxiety-like behavior and, at high doses or in particular pathological contexts (i.e., TBI), seizures [170]. Conversely, OX_2_-biased compounds exhibit a wider safety margin, producing smoother arousal with minimal cardiovascular load [24]. Still, they may disrupt nocturnal sleep if administered too late in the day, and chronic overstimulation could disrupt sleep architecture [171]. Importantly, the discontinuation of TAK-994 due to hepatotoxicity illustrates that the transition to clinical use is not risk-free and underscores the need to optimize hepatic safety in orally administered OX_2_ agonists as well as careful dose finding and long-term monitoring.

In systemic illness, metabolic demands must be carefully matched: in sepsis, early orexin enhancement improves survival and dampens neuroinflammation in animal models, but if applied before hemodynamic stabilization it may increase oxygen consumption and exacerbate tissue stress [154]. Chronic orexin-A supplementation has worsened anxiety-like behavior and cognitive outcomes in at least one repetitive mild TBI model, suggesting that prolonged or excessive stimulation may interfere with adaptive circuit reorganization rather than supporting it.

Taken together, these observations indicate that orexin-targeted therapies must be deployed with careful attention to phase, dose, receptor selectivity, and systemic status. Early-phase interventions need to balance arousal rescue and cytoprotection against cardiovascular and metabolic load, whereas subacute and chronic approaches should prioritize circadian alignment, avoidance of nocturnal hyperarousal, and long-term organ safety. Dose titration, individualized scheduling, and close monitoring of cardiovascular, metabolic, and sleep parameters will be indispensable for safe translation.

## 8. Limitations of the Current Evidence

Although orexinergic modulation shows conceptual promise across acute CNS injuries, several limitations constrain the strength and generalizability of the current evidence. Methodologically, both preclinical and clinical studies exhibit substantial heterogeneity in injury models, timing of orexin measurements, dosing protocols, behavioral endpoints, and routes of administration. In clinical cohorts, orexin levels are often obtained at single time points and rarely longitudinally, and many studies cannot fully control for comorbidities, concurrent medications, or systemic complications that influence arousal, inflammation, or metabolism, complicating interpretation of biomarker–outcome relationships [84,95,96].

Mechanistically, important gaps remain regarding receptor-specific contributions. Many studies do not isolate OX_1_- versus OX_2_-mediated effects, even though these receptors subserve distinct arousal, autonomic, and immune functions with clear therapeutic implications [24,97,98]. This ambiguity limits the rationale for receptor-selective interventions, as cytoprotective effects often attributed to OX_1_ and arousal-stabilizing actions attributed to OX_2_ are not always experimentally disentangled [102,155,160]. Although several studies implicate orexin in inflammatory, vascular, or stress-related cascades [106,111,117], many pathways remain based on indirect readouts rather than receptor-resolved mechanistic analyses.

Significant constraints also apply to therapeutic strategies themselves. Orexin-A peptides exhibit short half-life, uncertain central bioavailability, and practical barriers for clinical use outside tightly controlled settings [54,56]. Small-molecule OX_2_ agonists remain largely untested in preclinical models of acute injury and are available only in restricted research contexts, with ongoing safety optimization limiting broad applicability [8,24]. Neuromodulation approaches such as VNS, MNS, DBS, or LIFUS show orexin engagement in selected traumatic models [134,139,140], but evidence remains sparse, and their relevance outside TBI has yet to be demonstrated.

A further limitation arises from the strong state-, circadian-, and behavior-dependence of orexin physiology. Orexin neuron activity varies across the sleep–wake cycle, environmental conditions, and stress states, meaning that both biomarker measurements and therapeutic effects may depend on factors not consistently reported or controlled in existing studies [5,24]. Such variability complicates the identification of reproducible therapeutic windows and may contribute to inconsistencies across studies measuring orexin-A in stroke, hemorrhage, or sepsis.

Finally, a critical gap across the literature is the near absence of sex-specific analyses. The orexin system exhibits known sexual dimorphism in baseline activity and physiological integration [4], and the pathophysiological trajectories of stroke, TBI, sepsis, and SCI are also sex-dependent. Yet most experimental studies either include a single sex or do not report stratified outcomes, limiting the generalizability of current findings and potentially obscuring sex-dependent variability in therapeutic response.

## 9. Conclusions and Future Perspectives

Acute injuries to the CNS, whether vascular, traumatic, or systemic, share a fundamental biological logic: an abrupt collapse of arousal and autonomic stability, rapid neuroimmune activation, and progressive disruption of network function, followed by slow and often incomplete attempts at circuit repair. Across this continuum, the orexin system operates as a dynamic regulator of vigilance, stress resilience, autonomic balance, and motivational drive. Evidence from experimental models shows that early orexin suppression parallels loss of arousal and autonomic instability, while its later recovery accompanies restoration of barrier integrity, motivation, and motor engagement. Controlled enhancement of this signaling can therefore influence not only survival but the quality and pace of functional recovery, though these effects remain highly dependent on injury phase, circuit state, and behavioral or circadian context.

Translational progress, however, is still uneven. The biological rationale for orexinergic intervention is strongest where arousal, vigilance, and motivational deficits dominate: post-stroke or post-traumatic fatigue, chronic hypoarousal after sepsis or cardiac arrest, and disorders of vigilance emerging in the subacute or chronic stages. These indications align with the current toolset of OX_2_-selective small-molecule agonists, already in human trials for sleep–wake disorders and adaptable to circadian-timed dosing. By contrast, early acute cytoprotection in focal ischemia, hemorrhage, or spinal trauma remains insufficiently defined: most preclinical studies test a single intervention time point and do not resolve whether very early orexin stimulation is stabilizing or potentially disruptive. Existing studies typically test a single time point and do not resolve whether very early orexin stimulation is consistently stabilizing or potentially disruptive. Importantly, no orexin-enhancing intervention has yet been evaluated clinically in any acute CNS injury, and existing human data remain limited to associations between CSF orexin levels and vigilance outcomes. The near-complete absence of long-term preclinical studies further limits translation; most investigations end within hours to days, leaving the chronic trajectory—and its therapeutic opportunities—essentially unmapped. Key gaps also persist in understanding how sex, age, comorbidities, and concurrent treatments shape orexinergic responsiveness across injury contexts.

A critical bottleneck is the lack of selective OX_1_ agonists and balanced dual agonists. Much of orexin’s cytoprotective, anti-inflammatory, and autonomic-stabilizing potential appears to depend on OX_1_ engagement, yet pharmacological tools remain almost entirely restricted to OX_2_-selective agents. This asymmetry narrows the therapeutic landscape and complicates attempts to target early injury mechanisms, where OX_1_-driven pathways may be most relevant.

Future work should therefore follow a two-track strategy: cautious clinical exploration in arousal-related syndromes using available OX_2_ agents, and deeper mechanistic and pharmacological research in acute injury models to delineate safe therapeutic windows and receptor-specific contributions. Longitudinal, sex-balanced, and multimodal preclinical designs will be essential to define therapeutic windows, chronic safety, and potential interactions with rehabilitation, neuromodulation, and metabolic support. Integrating orexinergic modulation with rehabilitation, circadian entrainment, and neuromodulatory or behavioral activation strategies may convert conceptual plausibility into measurable functional benefit. This requires multidisciplinary collaboration, bridging neuropharmacology, chronobiology, and clinical neuroscience to transform conceptual plausibility into therapeutic reliability.

In summary, orexinergic modulation offers a biologically grounded strategy to bridge acute neuroprotection with long-term functional recovery. Rather than a single-target intervention, orexin signaling represents a flexible, state-sensitive axis that links arousal, vigilance, and motivated behavior throughout the recovery process. By aligning future orexin-based interventions with the temporal, sex-specific, and circuit-level dynamics that characterize CNS repair, it may be possible to shorten the trajectory from survival to functional independence, redefining how post-injury repair is understood and pursued.

## Figures and Tables

**Table 1:** Strategies to enhance orexinergic tone for therapeutic application after acute CNS injury. Summary of pharmacological, physiological, and neuromodulatory approaches aimed at increasing central orexin signaling. Each strategy is characterized by its key features, stage of development, advantages, limitations, and anticipated utility across phases of acquired CNS injury. Abbreviations: OX_1_/OX_2_, orexin receptor 1/2; BBB, blood–brain barrier; iv, intravenous; PK, pharmacokinetics; BCAA, branched-chain amino acids; VNS, vagus-nerve stimulation; MNS, median-nerve stimulation; DBS, deep-brain stimulation; AAV, adeno-associated virus.

Strategy	Main Features	Development Stage	Advantages	Limitations	Potential in Acute CNS Injury	Risks/Concerns
Synthetic small-molecule agonists (OX_2_ selective)	Oral/iv, BBB-penetrant; strong wake-promoting effect	Late-stage clinical	Drug-like; scalable; predictable PK; circadian-timed dosing feasible	No OX_1_ activity; long-term safety unclear; hepatotoxicity concerns (TAK-994)	Best for subacute–chronic hypoarousal and rehab engagement	Insomnia; cardiovascular load; possible organ toxicity
Peptide replacement (orexin-A/B)	Intranasal delivery; dual-receptor engagement; CNS effects documented	Early translational	Rapid onset; strong biological effects; non-invasive	Short half-life; variable CNS uptake; possible peripheral mediation; supraphysiologic doses	Acute arousal rescue; brief pre-rehab priming	Tachycardia; inconsistent bioavailability; desensitization
Endogenous modulation (diet/behavior)	Exercise, BCAA, thermogenic and circadian cues	Preclinical/observational	Accessible; low-cost; scalable; aligns with rehabilitation	Mechanistically nonspecific; state-dependent; variable effects	Supports rehab motivation, circadian stabilization	Limited in highly impaired individuals
Neuromodulation (MNS, VNS, DBS)	Circuit-level stimulation; indirect orexin recruitment	Experimental/clinical (for other indications)	Bypasses PK limits; enhances arousal and autonomic stability	Invasive (DBS/VNS); protocols not standardized; access limited	Acute arousal, subacute circuit re-engagement, chronic rehab reinforcement	Autonomic fluctuations; surgical risks; off-target effects
Cell/gene therapy replacement	Orexin grafts; AAV orexin re-expression; effective in narcolepsy models	Preclinical/translational (for narcolepsy)	Potential long-term restoration of orexin tone	Invasive; not suited for secondary dysfunction; cost and limited access	Currently no realistic application; future indirect uses possible	Long-term safety unclear: immune rejection; tumorigenesis risks

**Table 2:** Main clinical studies reporting orexinergic alterations or interventions in models of acute CNS injury and systemic inflammatory or metabolic insults. ↓, decreased level or effect; ↑, increased level or effect. Abbreviations: BBB, blood–brain barrier; CSF, cerebrospinal fluid; dpi, days post-injury; ICH, intracerebral hemorrhage; mpi, months post-injury; SAH, subarachnoid hemorrhage; TBI, traumatic brain injury; ypi, years post-injury.

Condition	N	Assessment Phase	Primary Outcomes	References
Ischemic stroke	1 patient	Chronic phase (5 ypi)	↓ CSF orexin-A	[83]
	29 patients 13 controls	Acute phase (2–3 dpi)	↓ CSF and serum orexin-A, infarct volume inversely correlated with CSF orexin-A but not with serum orexin-A	[84]
	163 patients	Acute phase (2–9 dpi)	↑ serum orexin-A predictive of short-term neurological improvement. Excess of orexin A associated with post-stroke insomnia	[85]
	183 patients	Acute phase (1–30 dpi)	↓ CSF orexin-A predictive for poor sleep quality after stroke	[86]
ICH	11 patients	Acute phase (0–13 dpi)	↓ CSF orexin-A	[87]
SAH	15 patients 5 controls	Acute phase (0–14 dpi)	CSF orexin-A levels correlated to consciousness	[88]
	15 patients	Acute phase (0–10 dpi)	↓ CSF orexin-A, especially with vasospasm and delayed neurological deficit	[89]
SAH and ICH	9 patients 12 controls	Acute/subacute phase (2–36 dpi)	↓ CSF orexin-A	[90]
TBI	44 patients 20 controls	Acute phase (1–4 dpi)	↓ CSF orexin-A	[91]
	65 patients	Chronic phase (6 mpi)	↓ CSF orexin-A in patients with post-TBI excessive daytime sleepiness	[92]
	4 patients 4 controls	Post-mortem (7–42 dpi)	↓ orexin neurons; ↑ gliosis	[93]
TBI and stroke	20 patients	Acute phase (0–14 dpi)	↓ CSF orexin-A predictive of poor outcome/death	[94]
Sepsis	1 patient	Acute phase (0–20 dpi)	↓ CSF orexin-A (restored when systemic inflammation ended), ↓ BBB integrity (orexin-A leakage)	[95]
Meningitis and encephalitis	14 patients	Acute phase (1 dpi)	↓ CSF orexin-A	[96]

**Table 3:** Summary of preclinical studies reporting orexinergic alterations or interventions in models of acute CNS injury and systemic inflammatory or metabolic insults. ↓, decreased level or effect; ↑, increased level or effect. Abbreviations: 4-Vo, four-vessel occlusion; ACA, asphyxial cardiac arrest; Akt, protein kinase B; BCAA, branched-chain amino acids; BBB, blood–brain barrier; BDNF, brain-derived neurotrophic factor; BCAO, bilateral common carotid artery occlusion; CCAO, common carotid artery occlusion; CCI, controlled cortical impact; CLP, cecal ligation and puncture; COX2, cyclooxygenase-2; CNS, central nervous system; dpi, days post-injury; EEG, electroencephalogram; eNOS, endothelial nitric oxide synthase; ERK, extracellular signal-regulated kinase; Glu, glutamate; HIF-1α, hypoxia-inducible factor 1α; HO-1, heme oxygenase 1; HR, heart rate; hpi, hours post-injury; icv, intracerebroventricular; ict, intracisternal; ICH, intracerebral hemorrhage; iNOS, inducible nitric oxide synthase; ip, intraperitoneal; iv, intravenous; LF/HF, low frequency/high frequency heart rate variability ratio; LH-DBS, lateral hypothalamus deep brain stimulation; LIFUS, low-intensity focused ultrasound; LPS, lipopolysaccharide; MAPK, mitogen-activated protein kinase; MCAO, middle cerebral artery occlusion; MEKK, MAPK/ERK kinase kinase; MNS, median nerve stimulation; mpi, months post-injury; N/A, not applicable; NF-κB, nuclear factor kappa B; NLRP3, NOD-, LRR- and pyrin domain-containing protein 3; NO, nitric oxide; NREM, non-rapid eye movement sleep; Nrf-2, nuclear factor erythroid 2-related factor 2; OX_1_/OX_2_, orexin receptor 1/2; p-mTOR, phosphorylated mammalian target of rapamycin; PAG, periaqueductal gray; PI3K, phosphoinositide 3-kinase; RAS, rat sarcoma; REM, rapid eye movement sleep; ROS, reactive oxygen species; RR, respiratory rate; SAH, subarachnoid hemorrhage; SCI, spinal cord injury; sc, subcutaneous; tPCS, transcranial pulsed current stimulation; TBI, traumatic brain injury; TLR4, Toll-like receptor 4; TNFα, tumor necrosis factor α; TNS, trigeminal nerve stimulation; VNS, vagus nerve stimulation.

Disease Context	Model/Condition	Intervention	Assessment Phase	Primary Outcomes	References
Ischemic stroke	CCAO MCAO	N/A	Acute phase (1–7 dpi)	↑ cell death ↑ OX_1_ expressed in neurons, astroglia and oligodendroglia within lesion core	[97,98]
	BCAO MCAO 4-Vo	icv orexin-A once pre- or post-injury	Acute phase (1–9 dpi)	Orexin-A ameliorates stroke consequences: ↓ infarct volume, ↓ neurological deficit, ↓ pain, ↑ blood pressure, flow and heart rate Neuroprotection: ↓ apoptosis, ↓ autophagy in vitro and in vivo (via ↓ p-ERK1/2 and ↑ p-mTOR), ↑ OX_1_, ↑ HIF-1α, eNOS, NO and BDNF expression, ↓ endothelin-1 expression ↓ neuroinflammation: ↓ excessive activation of astrocytes, ↓ pro-inflammatory cytokines, NF-κB p65 pathway and MAPK pathway	[99,100,101,102,103,104,105,106,107]
	MCAO CCAO	Sleep deprivation 6 h pre-injury	Acute phase (3–7 dpi)	Sleep deprivation ameliorates stroke consequences: ↓ infarct volume, ↑ REM ↓ upregulation of genes related to immune response and cell division	[108]
	MCAO	Orexin overexpression plasmid 3 days before MCAO	Acute/Subacute phase (1–10 dpi)	Plasmid ameliorates stroke consequences: ↓ infarct volume, ↑ sleep structure and neurological function (via Glu uptake and GABA levels) Neuroprotection: ↓ neuronal apoptosis	[109]
	MCAO	IV parecoxib (COX2 inhibitor)	Acute phase (3 dpi)	Parecoxib ameliorates stroke consequences, and ↑ orexin positive cells and orexin levels	[110]
Hemorrhagic stroke	Injection of autologous blood into basal ganglion (ICH)	intranasal or icv orexin-A once post-injury	Acute/Subacute/ Chronic phase (1–28 dpi)	Orexin-A ameliorates ICH consequences: ↓ neurological deficit, ↓ brain water content Neuroprotection: ↓ neuronal damage, ↓ autophagy (via ERK/mTOR pathway), ↑ p-CaMKKβ, p-AMPK, and anti-inflammatory cytokines, ↓ p-NF-κB and pro-inflammatory cytokines	[111,112]
Cardiac arrest	ACA Major cardiac vessels compression (transient global ischemia)	N/A	Acute phase (0–7 dpi)	↑ cell death. Better neurological function correlated with ↑ HR post-resuscitation, ↑ LF/HF ratio and ↑ gamma band power ↑ CSF orexin-A at 24 hpi but ↓ CSF orexin-A at 2 and 4 dpi	[113,114]
	ACA	intranasal or icv orexin-A once post-injury	Acute (0–3 dpi) and Subacute phase (12 dpi)	Orexin-A meliorates ACA consequences: ↓ neurological deficit, ↑ EEG entropy, ↑ arousal (EEG gamma fraction) ↑ OX_1_ expression ↓ neuroinflammation: ↓ TNFα, iNOS, CD11b	[115,116,117,118]
	ACA	ip suvorexant (OX_1_/OX_2_ antagonist) once or thrice (5 min, 10 h, 20 h) post-injury	Acute phase (0–3 dpi)	Suvorexant has detrimental effects: No neurological recovery, ↓ HR, ↓ LF/HF ratio	[119,120]
	ACA	Caloric restriction once overnight pre-injury	Acute phase (0–3 dpi)	Caloric restriction ameliorates ACA consequences: ↓ neurological deficit ↓ stress-induced hyperglycemia, ↑ blood ketone levels, no change in sirtuin-1 Neuroprotection: ↓ neurodegeneration, no change in BDNF	[121]
Hypoxia–Ischemia	Transient focal ischemia in vivo	Orexin-A once pre-injury	Acute phase	Orexin-A ameliorates hypoxia–ischemia consequences: ↓ infarct volume Neuroprotection: ↓ ROS accumulation and neuronal death, ↑ PI3K/Akt survival pathway	[122]
	Cobalt chloride on primary cortical neuronal cell culture	Orexin-A/B incubation for 24–48 h	Acute phase (1–2 dpi)	Orexin-A/B ameliorate hypoxia–ischemia consequences: Neuroprotection: ↑ neuronal viability (via Akt activation)	[123]
	Chemical hypoxia	Orexin-A/B incubation	Acute phase	Orexin-A/B ameliorate hypoxia–ischemia consequences: Neuroprotection: ↑ neuronal viability, ↓ oxidative stress (via MEKK and Akt activation)	[124]
TBI	CCI Fluid perfusion injury	N/A	Acute/Subacute phase (1–30 dpi)	↑ cognitive deficit, ↓ wakefulness, ↑ REM and NREM sleep, ↑ depressive-like symptoms, ↓ motor activity ↓ orexin-A, orexinergic neurons and orexinergic activity, ↑ astrogliosis	[125,126,127,128,129,130]
	Modified Feeney’s method	icv orexin-A once post-injury	Acute phase (0–3 dpi)	Orexin-A ameliorates TBI consequences: ↓ neurological deficit, ↓ lesion volume Neuroprotection: ↓ neuronal damage, ↓ ferroptosis (via Nrf2/HO-1 pathway) ↓ pro-inflammatory cytokines	[131]
	CCI	DORA (dual orexinergic antagonist) once post-injury	Acute/Subacute/ Chronic (7 dpi–3 mpi)	Acute orexinergic inhibition contributed to TBI consequences: ↑ sleep fragmentation, ↑ sleepiness	[132]
	CCI	oral gavage DORA-22 (dual orexin antagonist) daily for 30 days post-injury	Subacute/Chronic phase (7 dpi–3 mpi)	Chronic orexinergic inhibition ameliorates TBI consequences: ↓ posttraumatic seizures, ↑ sleep homeostatic drive ↑ GABAergic inhibition (related to seizures)	[133]
	CCI	tPCS post-injury	Acute phase (1–7 dpi)	tPCS ameliorates TBI consequences: ↓ neurological, motor and cognitive deficit ↑ orexin-A Neuroprotection: ↓ tissue damage All effects are OX_1_-dependent	[72]
	Free fall drop/ Modified Feeney’s method	VNS post-injury MNS post-injury TNS post-injury	Acute phase (1 dpi)	VNS, MNS and TNS ameliorate TBI consequences: ↑ consciousness (via ↑ RasGRF1 pathway), ↓ neurological deficit, ↓ brain edema ↑ orexin-A and OX_1_ expression, Neuroprotection: ↓ brain cellular and tissue damage, ↓ neuronal pyroptosis (via ↓ NLRP3/Caspase-1/gasdermin D) ↓ pro-inflammatory cytokines, ↓ TLR4/NF-κB/NLRP3 inflammasome All effects are OX_1_-dependent	[73,134,135,136,137,138]
	Free fall drop	LH-DBS post-injury	Acute phase (12 hpi)	LH-DBS ameliorates TBI consequences: ↑ consciousness ↑ orexin-A/OX_1_ expression All effects are OX_1_-dependent	[139]
	Free fall drop	LIFUS daily for 3 days post-injury	Acute phase (3 dpi)	LIFUS ameliorates TBI consequences: ↓ neurological deficits, ↓ brain edema ↑ orexin-A and OX1 expression Neuroprotection: ↓ tissue damage, ↓ necrotic neuronal degeneration ↓ pro-inflammatory cytokines, ↓ NF-κB/NLRP3 inflammasome	[140]
	Fluid percussion injury	BCAA dietary supplementation from day 2 to 7 post-injury	Acute/Subacute/ Chronic (4–30 dpi)	BCAA ameliorates TBI consequences: ↑ wakefulness, ↓ fragmented wake bouts ↑ Orexin neurons activation (via ↑ glutamatergic presynaptic density)	[70,141]
	Repeated mild TBI	Chronic icv orexin-A daily from day 5 to 33 post-injury	Chronic phase (14–28 dpi)	↓ orexin+ neurons, ↓ orexinergic projections to PAG, ↑ nociceptive sensitivity, ↓ anxiety-like behavior Orexin-A worsened outcomes: ↑ anxiety and motor deficits, ↑ CSF metabolomic disruption	[142,143]
SCI	Complete spinal cord transection at T9	intrathecal orexin-A daily for 3 days post-injury	Acute phase (1–7 dpi)	Orexin-A ameliorates SCI consequences: ↑ motor recovery ↓ overactive/dysregulated Glu dynamics	[144]
Systemic biological and toxic insults	ip LPS in wildtype and/or orexin-ablated (OX/AT3 transgenic) mice	N/A	Acute phase (1–3 dpi)	↓ gait speed and exploration, ↑ NREM sleep, ↓ wakefulness ↓ CSF orexin-A levels, ↓ orexin neurons, ↓ prepro-orexin expression, ↓ Fos expression in orexin neurons ↑ cytokines In orexin-ablated mice: ↓ survival	[96,145,146,147,148,149,150,151,152]
	Acute pancreatitis (non-infectious)	N/A	Acute phase (1 dpi)	↑ brain levels of β-endorphin, orexin, and oxytocin Neuropeptide upregulation occurred before local cytokine increase or microglial activation	[153]
	Cecal ligation and puncture	intranasal or icv Orexin-A once or daily for 7 days post-injury	Acute phase (1–7 dpi)	Orexin-A ameliorates sepsis consequences: ↑ survival, ↓ cognitive and emotional deficit, ↓ brain edema, ↑ responsiveness, ↑ pituitary function ↓ tissue damage, ↓ BBB disruption, ↓ neuroinflammation: ↓ pro-inflammatory cytokines, ↓ microglia activation Effects mediated via OX_2_ and RAS/MAPK pathway	[154,155]
	Cecal ligation and puncture	ip xanomeline (mAChR agonist) thrice (0, 23, 47 h) post-injury	Acute phase (2 dpi)	Xanomeline ameliorates sepsis consequences: ↑ orexinergic activity, ↑ temp, HR and RR ↑ pituitary function ↓ pro-inflammatory cytokines Effects reversed by OX_1_/OX_2_ antagonist (almorexant)	[156]
	LPS-induced endotoxemia	ict, ip or sc orexin-A once pre- during or post-injury	Acute phase (1–3 dpi)	Central, peripheral orexin-A (crosses BBB in endotoxemia): ↑ survival restores body temperature, cardiovascular tone ↓ cytokine levels ↑ catecholamines, ↑ corticosterone Effects blocked by OX_1_ antagonist, vagotomy, or atropine	[157,158]
	Alcohol-induced coma	icv orexin-A/B once post-injury	Acute phase (7 dpi)	Orexin-A/B ameliorates intoxication consequences: ↓ coma duration, restores righting reflex ↓ delta power in EEG, ↑ prefrontal cortex activity	[159]

## Data Availability

No new data were created or analyzed in this study. Data sharing is not applicable to this article.
